# Genetic Underpinnings of Host Manipulation by *Ophiocordyceps* as Revealed by Comparative Transcriptomics

**DOI:** 10.1534/g3.120.401290

**Published:** 2020-04-30

**Authors:** Ian Will, Biplabendu Das, Thienthanh Trinh, Andreas Brachmann, Robin A. Ohm, Charissa de Bekker

**Affiliations:** * Department of Biology, College of Sciences, University of Central Florida, Orlando, Florida, 32816,; ^†^ Genetics, Faculty of Biology, Ludwig-Maximilians-University Munich, Martinsried, Germany, 82152, and; ^‡^ Microbiology, Department of Biology, Faculty of Science, Utrecht University, The Netherlands, 3584

**Keywords:** Parasitic manipulation, molecular host-parasite interactions, fungi, ants

## Abstract

Ant-infecting *Ophiocordyceps* fungi are globally distributed, host manipulating, specialist parasites that drive aberrant behaviors in infected ants, at a lethal cost to the host. An apparent increase in activity and wandering behaviors precedes a final summiting and biting behavior onto vegetation, which positions the manipulated ant in a site beneficial for fungal growth and transmission. We investigated the genetic underpinnings of host manipulation by: (*i)* producing a high-quality hybrid assembly and annotation of the *Ophiocordyceps camponoti-floridani* genome, (*ii*) conducting laboratory infections coupled with RNAseq of *O. camponoti-floridani* and its host, *Camponotus floridanus*, and (*iii*) comparing these data to RNAseq data of *Ophiocordyceps kimflemingiae* and *Camponotus castaneus* as a powerful method to identify gene expression patterns that suggest shared behavioral manipulation mechanisms across *Ophiocordyceps*-ant species interactions. We propose differentially expressed genes tied to ant neurobiology, odor response, circadian rhythms, and foraging behavior may result by activity of putative fungal effectors such as enterotoxins, aflatrem, and mechanisms disrupting feeding behaviors in the ant.

Transmission from one host to the next is a crucial step in the life cycle of parasites. Certain parasites have evolved to adaptively manipulate the behavior of their animal hosts to aid transmission. Many examples of manipulating parasites and their hosts have been reported across taxa and are active topics of research (Moore 1995, 2013; Thomas *et al.* 2010; Lafferty and Kuris 2012; Poulin and Maure 2015; de Bekker *et al.* 2018; Hafer-Hahmann 2019), with the ant-manipulating *Ophiocordyceps* fungi emerging as a notable model (de Bekker *et al.* 2014a; de Bekker 2019). However, in most parasitic manipulation systems, including *Ophiocordyceps*-ant interactions, the mechanisms by which the parasite dysregulates animal behavior are largely unknown (Herbison 2017). As such, the study presented here seeks to home in on the major players involved in *Ophiocordyceps* infection and manipulation of carpenter ants by using a comparative transcriptomics framework to identify, compare, and discuss candidate genes underlying manipulation across two different fungus-ant species interactions. The species we compare are *Ophiocordyceps kimflemingiae* and its host *Camponotus castaneus*, on which mechanistic work has previously been performed (de Bekker *et al.* 2014b 2015; Fredericksen *et al.* 2017; Mangold *et al.* 2019; Loreto and Hughes 2019), and *Ophiocordyceps camponoti-floridani* (Araújo *et al.* 2018) and its host *Camponotus floridanus*. The interactions between the latter pair have not yet previously been investigated.

Ant-manipulating *Ophiocordyceps* infect ants and modify their behavior to complete the parasite life cycle, at a lethal cost to the host. Infected ants display hyperactivity or enhanced locomotor activity (ELA) (Hughes *et al.* 2011; de Bekker *et al.* 2015), deviation from foraging trails (Pontoppidan *et al.* 2009; Hughes *et al.* 2011), and a summiting behavior coupled with biting and clinging to attach themselves to vegetative substrates until death (Pontoppidan *et al.* 2009; Andersen *et al.* 2009; Hughes *et al.* 2011; Mongkolsamrit *et al.* 2012; Chung *et al.* 2017; Andriolli *et al.* 2019; Loreto *et al.* 2018). This final fatal change in behavior is the most tractable readout for manipulation of the host, and provides a growth and transmission site that appears to be adaptive for the fungal parasite (Andersen *et al.* 2012; Loreto *et al.* 2014). Bioactive compounds with neuromodulatory and physiology-disrupting effects (de Bekker *et al.* 2014b 2015; Kobmoo *et al.* 2018; de Bekker 2019; Loreto and Hughes 2019), and tissue destruction and hypercontraction of jaw muscles (Hughes *et al.* 2011; Fredericksen *et al.* 2017; Mangold *et al.* 2019) have been proposed as possible means of dysregulating host behavior. Moreover, manipulated biting appears to be synchronized by time of day in multiple *Ophiocordyceps*-ant species interactions (Hughes *et al.* 2011; de Bekker *et al.* 2014a, 2015, 2017b; de Bekker 2019). This suggests that *Ophiocordyceps* fungi also employ mechanisms to modify host behaviors that operate according to daily rhythms and are under control of the hosts’ biological clocks (Hughes *et al.* 2011; de Bekker *et al.* 2014a, 2015, 2017b; de Bekker 2019).

Multiple reports indicate that manipulation of ant behavior only occurs in a host-specific manner, with a single species of *Ophiocordyceps* manipulating a single species of ant (Evans *et al.* 2011; de Bekker *et al.* 2014b; Araújo *et al.* 2018; Sakolrak *et al.* 2018). Part of the mechanisms involved in manipulation of ant behavior might, therefore, be species specific (de Bekker *et al.* 2017a). However, convergently evolved and conserved mechanisms are likely also shared among these specialized *Ophiocordyceps* fungi as they have common evolutionary histories (Araújo and Hughes 2019) and are confronted with similar ecological obstacles (*i.e.*, the modification of ant behavior to attach to elevated transmission sites) (Chetouhi *et al.* 2015; Loreto *et al.* 2018). Investigating these shared mechanisms across *Ophiocordyceps* and their host ant species would elucidate the common elements involved and aid in the identification of candidate genes and compounds that are key to establishing manipulation. Comparative proteomics to understand manipulated phenotypes induced by mermithid worms have demonstrated that such approaches can identify candidate convergent mechanisms of host manipulation across taxa (Herbison *et al.* 2019). As such, we conducted comparative transcriptomics to reveal candidate genes underscoring manipulation that could offer new insights into ant neurobiology and behavior, novel fungal bioactive compounds, and specific understanding of *Ophiocordyceps-Camponotus* interactions. These comparative studies may provide evidence and hypotheses for molecular mechanisms driving comparable manipulation phenotypes in other systems. Baculoviruses that manipulate the behavior of moth larvae also elicit host ELA, climbing, and eventual death at an elevated position, thereby dispersing viral propagules (Kamita *et al.* 2005; van Houte *et al.* 2014a; Han *et al.* 2018). Two primary viral genes have been proposed to be necessary in driving manipulation in this system, ecdysteroid UDP‐glucosyl transferase (*egt*) (Hoover *et al.* 2011; Ros *et al.* 2015; Han *et al.* 2015) and protein tyrosine phosphatase (*ptp*) (Kamita *et al.* 2005; Katsuma *et al.* 2012). Another example of a fatal summiting phenotype as a result of parasitic manipulation is induced by the distantly related *Entomophthora muscae* fungi that infect and manipulate flies (Krasnoff *et al.* 1995; Elya *et al.* 2018). Since *Ophiocordyceps*, Baculovirus and *Entomophthora* are all presented with the similar challenge of inducing summiting behavior to establish effective parasite transmission, quite plausibly their manipulation mechanisms have convergently evolved.

In the study presented here, we infected *C. floridanus* with *O. camponoti-floridani* and performed RNAseq on both organisms sampled before infection, during manipulated clinging, and after host death. Subsequently, we compared our gene expression data to previous transcriptomics work done in *O. kimflemingiae* and *C. castaneus* (de Bekker *et al.* 2015). Both *Ophiocordyceps* species reside in different clades within the *Ophiocordyceps unilateralis* species complex (Araújo *et al.* 2018), which are genomically vastly different (de Bekker *et al.* 2017a). Furthermore, our approach informs RNAseq of *C. floridanus* with a corresponding and updated genome (Shields *et al.* 2018), unlike the previous work that was constrained to using a *C. floridanus* genome to inform RNAseq of *C. castaneus*. With this framework, we transcriptionally compare fungal parasites and ant hosts, highlighting possible shared mechanisms involved in manipulation. To this end, we also report the first annotated genome assembly of *O. camponoti-floridani* using a long-read short-read hybrid approach. We propose candidate fungal genes and possible scenarios by which they may contribute to infection and manipulation of *Camponotus* hosts. Similarly, we highlight host genes that possibly reflect changes in behavior and challenges to physiology due to fungal infection and manipulation. Our findings include changes in genes associated with ant neurobiology, odor detection, and nutritional status, as well as fungal genes related to toxins, host feeding behavior pathways, proteases, and putative effectors similar to those previously reported in Baculovirus. We propose possible scenarios by which these genes reflect or promote changes in host behavior and physiology as further evidence or new grounds for hypotheses in the field of behavior manipulating parasitism. We have organized our findings and these scenarios in several comprehensive sections below that discuss principal component analyses (PCA) and weighted gene coexpression network analyses (WGCNA) analyses for ant and fungal data, functional enrichments and differentially expressed genes found in both ant and fungus, and upregulated fungal secondary metabolite clusters.

## Materials And Methods

### Fungal isolation & culture

To sequence the genome and perform infection studies followed by transcriptome sequencing, we isolated and cultured two strains of the fungus *Ophiocordyceps camponoti-floridani*. Strain EC05, used for the *de novo* genome assembly, was collected from Little Big Econ State Forest in Seminole County, Florida. Strain Arb2 was collected at the University of Central Florida arboretum in Orange County, Florida and used for laboratory infections and RNAseq data. These samples were obtained by permission from the University of Central Florida and the Florida Department of Agriculture and Consumer Services.

Both strains were isolated by surface sterilizing infected *Camponotus floridanus* cadavers in 70% ethanol for 10 sec and aseptically removing ant cuticle with 25 G needles (PrecisionGlide, BD) to extract *O. camponoti-floridani* mycelium. Extracted fungal masses were plunged into a solid medium (7.8 g/L potato dextrose agar [BD], 6 g/L agar [BD], 100 mg/L Penicillin/Streptomycin [Gibco], and 100 mg/L Kanamycin [Alfa Asear]) and maintained at 28° for 15 days to screen for possible contaminants and indications of sample viability. We placed viable extractions into liquid culture in T25 tissue flasks (CytoOne, USA Scientific) containing Grace’s Insect Medium (Unsupplemented Powder, Gibco) supplemented with 10% Fetal Bovine Serum (FBS) (Sterile Filtered US Origin, Gibco). Incubation at 28° and 50 rpm promoted blastospore growth. Once the culture was established, we reduced FBS to 2.5% for secondary cultures.

Both EC05 and Arb2 nuclear 18S ribosomal short subunit (SSU) sequences matched voucher JA-2017c Flx1 (Araújo *et al.* 2018) with 100% identity, confirming these strains as *O. camponoti-floridani*. We used SSU primers NS1 and NS4 (White *et al.* 1990), which yielded an approximately 1kb PCR amplicon with a Phusion High Fidelity Polymerase (New England Biolabs [NEB]) and the following PCR protocol: initial denaturation at 98° for 30 sec, 30 cycles of 98° for 10 sec, 49° for 30 sec, 72° for 30 s, and final elongation at 72° for 10 min.

### Whole genome sequencing and assembly

Strain EC05 was used to generate a high-quality draft genome for *O. camponoti-floridani* through a combination of Nanopore long-read and Illumina short-read sequencing. To extract DNA, we disrupted blastospore pellets frozen in liquid nitrogen with a 1600 MiniG tissue homogenizer (SPEX) at 1300 rpm for 30 sec. Samples were processed in 2 mL microcentrifuge tubes (Greiner) containing two steel ball bearings (5/32” type 2B, grade 300, Wheels Manufacturing) and kept frozen throughout disruption. We extracted DNA with 0.9 mL Extraction Buffer (1% SDS [Fisher Scientific], 240 mg/L para-aminosalicyclic acid [ACROS], 484 mg/L Tris/HCl [Fisher Scientific], 365 mg/L NaCl [Fisher Scientific], and 380 mg/L EGTA [MP] at pH 8.5) and 0.9 mL phenol/chloroform (Fisher Scientific). After phase separation, we washed the water phase with chloroform (Alfa Aesar) prior to extracting DNA with isopropanol. Following a 70% ethanol wash and reconstituting in nucleotide-free water (Gibco) we treated the DNA samples with RNase (Thermo Scientific).

A short-read DNA library was prepared with the Nextera DNA Flex Library Prep Kit (Illumina) with an average fragment length of 390 bp. Indexing for paired-end reads was performed with Nextera i5 and i7 adapters (Nextera Index Kit Index – 1 and 2). Short-read sequences were generated by sequencing 300 bp paired-end reads on an Illumina MiSeq (v 3, Miseq Reagent Kit) at the Genomics Service Unit (LMU Biocenter), resulting in 8 GB of fastq data. Reads were then quality filtered and adapter trimmed using BBduk (Bushnell 2019) as a plugin through Geneious Prime (v 2019.0.3, Biomatters) (trimq = 15, minlength = 75).

To facilitate long-read sequencing, we first size selected genomic DNA for fragments longer than 5 kbp on a Blue Pippin (Sage Science) with 0.75% agarose and a High-Pass protocol. A long-read library was subsequently generated using the SQK-LSK109 Ligation Sequencing Kit (Oxford Nanopore) according to manufacturer’s protocols. Sequencing on a PromethION (R9 flowcell, Oxford Nanopore) at the Laboratory for Functional Genome Analysis (LMU Gene Center) generated 105 GB (estimated 180x coverage) of Nanopore sequence data. Sequencing reads were base called with Albacore (v 2.2.5, Oxford Nanopore) and adapters were trimmed with Porechop (Wick 2018). We assembled the initial long-read genome using Canu (v 1.7.1, genomeSize = 45m, default settings) (Koren *et al.* 2017). An overestimation of the genome size allowed us to generate an assembly with good coverage despite the presence of bacterial contaminants (see below). This initial Canu long-read assembly was polished using raw Nanopore read data through Nanopolish (v 0.10.2) (Loman *et al.* 2015), followed by Illumina reads (120x coverage) with three iterations of Pilon (Walker *et al.* 2014) (v 1.23,–fix all) to produce a hybrid assembly. We identified a putative mitochondrion contig by testing for circular sequence structure with Circlator (Hunt *et al.* 2015), MUMmer (Kurtz *et al.* 2004), and Canu (Koren *et al.* 2017).

The assembly contained bacterial contaminant contigs that we removed. We identified contaminant contigs by their: (*i*) low read coverage aligned with Minimap2 (Li 2018) using all EC05 reads (Nanopore average coverage: 553x of *O. camponoti-floridani* genome contigs and 58x of contaminant contigs, and, Illumina: 195x of genome contigs and 15x of contaminant contigs); (*ii*) low RNA coverage with HISAT2 (Kim *et al.* 2015) mapping of Arb2 RNAseq control culture samples (62x of *O. camponoti-floridani* genome contigs and 0.14x of contaminant contigs); and (*iii*) high mapping to known bacterial genomes, *Cohnella* sp. 18JY8-7 (GenBank CP033433.1), *Delftia acidovorans* isolate ANG1 (GenBank CP019171.1), and *Stenotophomonas maltophilia* strain ISMMS2 (GenBank CP011305.1) (0.08% overlap between *O. camponoti-floridani* genome contigs and these bacteria genomes, and 28.96% overlap of contaminant contigs with these bacteria genomes).

### Genome annotation

We predicted genes in the EC05 *O. camponoti-floridani* genome using Augustus (v 3.0.2) trained with BRAKER1 (v 1.1.8) and intron hints from Arb2 transcripts (Stanke *et al.* 2008; Hoff *et al.* 2016). Protein domains predicted by PFAM (v 32) (Finn *et al.* 2014) were used to identify associated GO terms (Ashburner *et al.* 2000; Hunter *et al.* 2009). Protease predictions were made with the MEROPS database and a BLASTp E-value cutoff of 1e-5 (Rawlings *et al.* 2014). We used TMHMM (v 2.0c) to annotate transmembrane domains (Krogh *et al.* 2001). Secretion signals were identified with SignalP (v 4.1) (Almagro Armenteros *et al.* 2019). We predicted small secreted proteins (SSPs) when genes were shorter than 300 amino acids, carried a SignalP secretion signal, and did not have a transmembrane domain outside the first 40 amino acids. We identified genes and clusters predicted to be involved in secondary metabolism using a pipeline based on SMURF (Khaldi *et al.* 2010; de Bekker *et al.* 2015), with parameter d = 3000 bp and parameter y = 6. Transcription factors were identified based on the presence of a PFAM domain with DNA-binding properties using PFAM mappings from (Park *et al.* 2008). For BLAST annotations, we used BLASTp (v 2.7.1) against the NCBI nr database to gather up to 25 hits with E-value ≤ 1e-3. For the final annotation, we passed these hits to the Blast Description Annotator of Blast2GO with default settings (Conesa *et al.* 2005). In addition to searches that returned no results, we considered descriptions starting with “hypothetical protein” or “predicted protein” to lack BLAST annotations. BLASTp searches of the predicted proteins of *O. camponoti-floridani* against the Pathogen-Host Interaction (PHI) database (Urban *et al.* 2017), mitochondrial proteins, fungal secondary metabolite cluster proteins, and the *O. kimflemingiae* genome were conducted using Geneious (v 2019.0.3, Biomatters) with E-value ≤ 1e-3 and bit-score ≥ 50.

We supplemented the published BLAST genome annotations of the latest version of the *C. floridanus* genome (v 7.5) (Shields *et al.* 2018) with PFAM and GO annotations using the InterPro database (Finn *et al.* 2017) through Blast2GO (Conesa *et al.* 2005). For these additional annotations, we used the longest transcript variant per gene. To allow for comparison of ant RNAseq results of our study to (de Bekker *et al.* 2015), we bridged the current *C. floridanus* assembly to the earlier version (v 1.0) (Bonasio *et al.* 2010) used by de Bekker *et al.* (2015) through BLASTp homology searches with Geneious (v 2019.0.3, Biomatters), taking the top hit after an E-value ≤ 1e-3 and bit-score ≥ 50 cutoff. Annotations from BLAST and further processing for submission to GenBank (NCBI) was done with gffutils (v 0.10.1, Daler) and table2asn (v 1.23.338, NCBI).

### Ant collection & husbandry

Ant infections and behavioral observations were done using a wild colony of *C. floridanus*. This colony was collected from the University of Central Florida arboretum in February 2018 and housed in the laboratory. The collected ants consisted of several hundred individuals including minors, majors, and brood. In order to acclimate the ants and entrain their biological clocks to laboratory conditions, we first subjected the colony to two days of constant light and constant 25° temperature in a climate controlled room. Following this clock “reset” we gave the colony three days of 12 hr – 12 hr light-dark cycles at 25° to entrain ants to light as a circadian zeitgeber (LD1212, lights begin at zeitgeber time ZT 0). During acclimation, the colony housed in a 9.5 L plastic container (42 cm long × 29 cm wide) lined with talcum powder (Fisher Scientific) and containing aluminum foil wrapped test-tubes (50 mL, Fisher Scientific) with moist cotton to serve as darkened, humid nest spaces. Ants fed *ad libitum* on 15% sucrose solution, autoclaved crickets, and water.

### Ant infections

For laboratory infections, we selected minor caste ants from the colony and housed them in two identical containers in each of two climate-controlled incubators. Incubator A (MIR-154, Panasonic) was programmable for light and temperature. Incubator B (I36VL, Percival) was programmable for light, temperature, and relative humidity (RH). Incubator A ran a program with LD1212 and 28° during the light phase and 20° during the dark phase. Incubator B maintained humidity at 70% RH, LD1212, and 28° to 20° temperature. The light phase of Incubator B included a 4hr increasing ramp step (ZT 0 dark and 20° transitioning to ZT 4 peak light and 28°), a 4hr peak light and temperature hold until ZT 8, and 4hr decreasing ramp step until ZT 12 (peak light and 28° to dark and 20°). Light, temperature, and humidity for both incubators were verified with a HOBO data logger (model U12, Onset, Bourne, MA) (Figure S1A-B). The incubators were not significantly different for survival of infected ants (*P* = 0.072, log-rank test). Therefore, we chose to consolidate all samples from these incubators for survival and RNAseq analysis.

Each container (33 cm × 22 cm) was lined with talcum and had a thin layer of playground sand on the bottom that we routinely moistened during observations to maintain an elevated humidity inside the ant enclosure. On one end, containers held a 50 mL Falcon tube (Corning) with moist cotton wrapped in aluminum foil and *ad libitum* 15% sucrose and water. On the opposite end, we placed two thin 12 cm high wooden sticks draped with locally collected “Spanish moss” (*Tillandsia usneodies*) and a single “air plant” (*Tillandsia spp*.) (Figure S1C). These plants are common natural substrates for manipulated ants to bite and cling to at local field sites.

We painted ants to distinguish treatment groups (POSCA paint pens, Uni) one to three days in advance of infection by fungal injection (de Bekker *et al.* 2014b). We injected ants without anesthesia using aspirator tubes attached to glass capillary needles (10 µL borosilicate capillary tubes, Fisher Scientific), pulled using a PC-100 Narishige instrument. Needle placement for injection was on the ventral side of the thorax, sliding under the prosternum. The night before injection, we removed sugar and water from ants to be infected to ease the procedure. We timed injections to begin at ZT 0 and not last more than 3.5 hr. Ants that survived the first 3 hr post-injection were placed into the experiment. Control ants were not injected. Sham treatment ants were injected with 1 µL of Graces-2.5% FBS. Infected ants were injected with 1 µL of 3.5 × 10^7^ blastospores/mL in Graces-2.5% FBS, harvested during log-phase growth (OD_660nm_ = 0.984, approximately 1.8 × 10^7^ cells/mL based on estimates with *Saccharomyces cerevisiae*). Blastospores were harvested immediately preceding injection, washed twice in deionized water, and re-suspended in Graces-2.5%FBS.

Incubator A contained 18 control, 13 sham, and 33 infected ants. Incubator B contained 12 control, 13 sham, and 30 infected ants. After 14 days post injection (dpi), we observed aggressive patrolling and cleanup of dead and dying ants. Therefore, we chose to separate infected ants from non-infected groups to reduce the chances of interference with cadavers or the progress of manipulation. Control ants and sham ants were removed from the experiment boxes and rehoused in similar containers directly next to their original box for the remainder of the experiment.

### Observations of infection progression and sample collection

We made daily observations for manipulated ant phenotypes and survival at ZT 0, 2, 4, 6, 8, and 23 with sporadic opportunistic surveys for manipulated ants. We additionally began observations at ZT 20 starting 18 dpi. We considered ants to be manipulated when they displayed clasping or biting onto any substrate. Individuals that ceased to move nor responded to agitation by air puffs were considered dead. Live manipulated ants collected for RNAseq were recorded as dead for survival analysis. We analyzed survival data using the R package survival (Therneau 2015) and visualized curves with survminer (Kassambara *et al.* 2019).

Upon visible behavioral manipulation, we froze whole-ant samples for RNAseq directly in liquid nitrogen. We sampled healthy live control ants at ZT 21, which corresponds to the time of observed manipulation in our study. Healthy controls, rather than sham-injected ants, were collected to better match the previous study on *O. kimflemingiae* and *C. castaneus*, which we reference for comparative transcriptomics (de Bekker *et al.* 2015). Upon flash freezing, we stored ants in pre-chilled 2 mL microcentrifuge tubes (USA Scientific) at -80° until RNA extraction. In total, we analyzed 13 ants for RNAseq: live manipulated n = 5, dead manipulated n = 5, healthy control n =3. To obtain fungal control samples of strain Arb2 (n = 3), blastospore cultures were harvested at ZT 21 after a constant light and 28° synchronization treatment for two days, followed by an entrainment period for five days at LD1212 and 28° to 20°. During this time, light and temperature of culture conditions were validated with a HOBO data logger (Figure S1B). Fungal control cultures were grown to a late-log phase (OD_660nm_ = 1.7) before harvesting by pelleting 1 mL of culture per sample and snap-freezing in liquid nitrogen. Any collections made during subjective dark were done under red-light (730 nm wavelength).

### RNAseq data generation and analysis

All frozen samples for RNAseq were disrupted in the same manner as fungal genomic DNA samples (see above) prior to RNA isolation. For ant samples, we first decapitated frozen cadavers in petri dishes chilled with liquid nitrogen and then proceeded to frozen tissue disruption using individual heads. We extracted RNA with a RNAqueous Micro kit (Life Technologies) according to the manufacturer’s protocol, without DNase treatment. We isolated mRNA with poly-A magnetic beads (NEB) from 500 ng total RNA for each sample. Subsequently, we converted purified mRNA to 300 bp fragment DNA libraries with the Ultra II Directional kit (NEB) and indexed samples for multiplexing (NEB).

All libraries were sequenced on an Illumina HiSeq as 100 bp single-end reads at the Laboratory for Functional Genome Analysis (LMU Gene Center), resulting in 27M to 56M reads for each sample. We trimmed reads using BBduk (Bushnell 2019) as a plugin through Geneious Prime (v 2019.0.3, Biomatters) to remove adapters and for quality (qtrim = rl, trimq = 10, minlength = 25). Our choice for a Q10 quality trim and minimum 25 bp length of RNAseq reads yielded a sufficient number and quality of reads while reducing risk of introducing biases from read processing (Williams *et al.* 2016).

**Figure 1:**
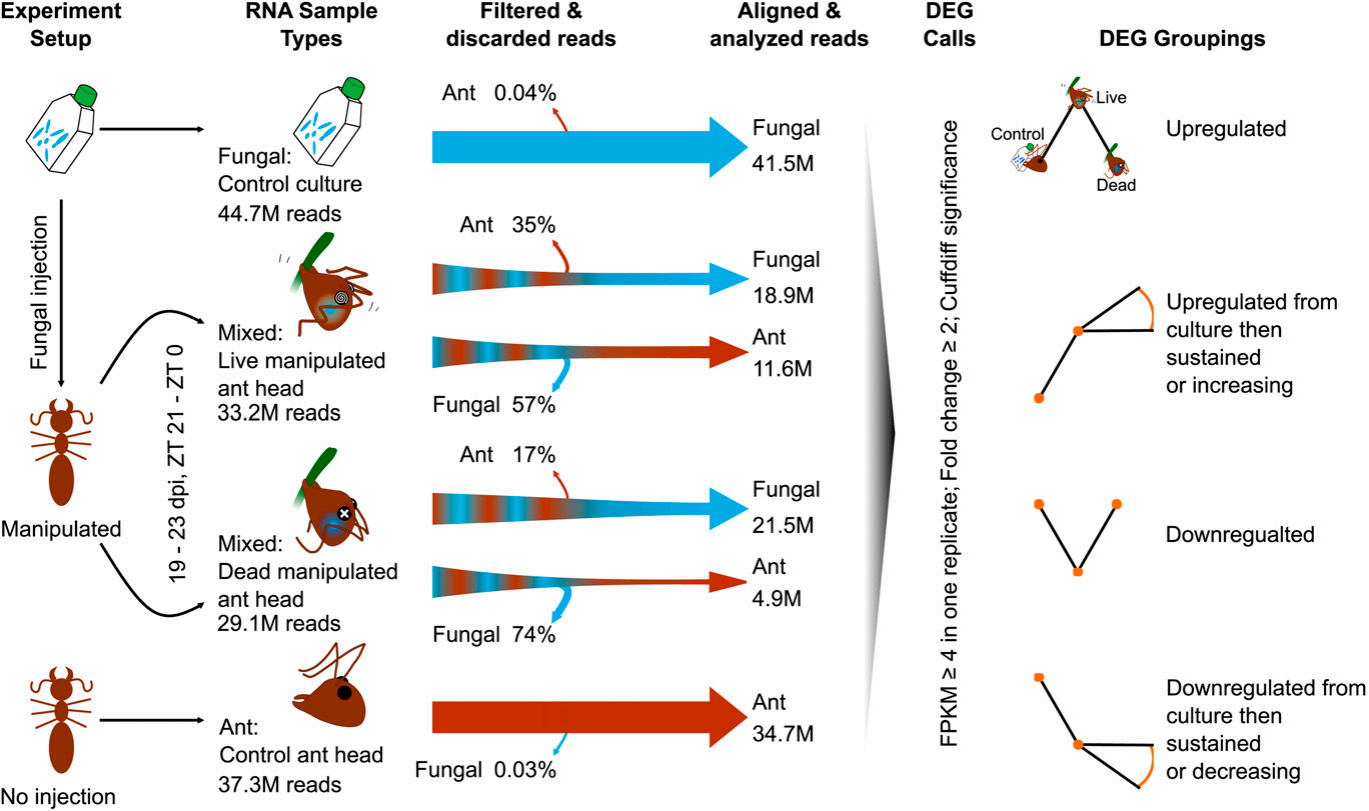
RNAseq experimental overview. Manipulated ant, ant control (no injection), and fungal control (blastospore culture) samples were processed for RNAseq. Manipulated ants contained mixed RNA of both the parasite and host. We observed and collected manipulation samples 19 – 23 dpi and between ZT 21 to ZT 0. Control ants and fungal culture samples were collected at ZT 21. To minimize possible bias from read counts of transcripts from mixed samples that map to either organism, we first filtered reads through either the host or parasite genome before aligning remaining reads to the other genome for gene expression analysis. DEGs were identified with Cuffdiff significance and expression cutoffs imposed for comparability to de Bekker *et al.* (2015). DEGs were then grouped by expression pattern over the course of the different sample points. All RNA samples taken from ants were extracted from whole heads. Read counts are the mean value per sample type.

For mixed transcriptome libraries (infected ants with host and parasite reads), we conservatively separated transcript sequences by first discarding all reads from the mixed sample that mapped to one organism’s genome before proceeding to analyze the other organism’s transcriptome (Figure 1). That is, we mapped to the host genome and then aligned the unmapped reads to the parasite genome, and *vice versa*. This method removes reads that map ambiguously to both the host and parasite from analysis. However, we estimate this to be only ≤ 0.04% of reads based on these organism’s transcriptomes in control conditions (Figure 1). All transcript mapping steps were done with HISAT2 (Kim *et al.* 2015).

We normalized and analyzed whole transcriptome gene expression levels with Cuffdiff with default settings (Trapnell *et al.* 2012). We used Cuffdiff significance calls (q ≤ 0.05, test = OK, significant = yes) to identify differentially expressed genes (DEGs) between sample groups. To consider DEGs biologically relevant to our analyses, we required genes to have an expression level of ≥ 4 RPKM in one replicate, and a minimum of twofold change between sample types. This methodology allows us to have the most comparability to published RNAseq data from *O. kimflemingiae* and *C. castaneus* (de Bekker *et al.* 2015)

We used unsupervised PCAs to describe the variation among control, live manipulated, and dead manipulated samples (R Core Team 2014; RStudio Team 2015). We ranked genes within a principal component (PC) by loading values to investigate the top 20 that explain the most variation within a PC. All gene RPKM transcription values were first log2(X+1) transformed for every gene with at least one sample replicate with RPKM ≥ 4. Plots were generated using R package ggplot (Wickham 2016).

Using a WGCNA we produced modules of coexpressed genes and associated them with control, live manipulated, and dead manipulated ants. Although our sample size is below an ideal replicate number, we applied this analysis for a coarse evaluation of possible gene modules associated with manipulation. Data were filtered (RPKM ≥ 4) and log2(X+1) transformed before analysis. We processed all samples together in the R package WGCNA (v 1.67) (Langfelder and Horvath 2008; R Core Team 2014; RStudio Team 2015). We applied a signed-hybrid network type, a soft power threshold equal to nine (fungus) or 12 (ant), minimum module size of 30, and default settings. Our categorical trait data were entered as either 0 (sample was not that type) or 1 (sample was that type) for control, live manipulation, and dead manipulated. For correlation of ant and fungal modules, eigengene values for ant modules were calculated with the moduleEigengenes function of the package and used as trait data for fungal module correlations. R package WGCNA was also used to generate a sample dendrogram to assess clustering of biological replicates (function hclust, with method = “average”).

We performed enrichment analyses on gene sets identified by PCA, WGCNA modules, and DEG groupings by using a hypergeometric test with Benjamini-Hochberg correction to correct for multiple testing (minimum number of genes with annotation term = 5, corrected p-value ≤ 0.05) (R Core Team 2014; RStudio Team 2015).

### Data availability

The version of the *Ophiocordyceps camponoti-floridani* genome assembly in this paper (JAACLJ010000000) has been deposited on Genbank under accession JAACLJ000000000. Read data for that assembly are available under BioProject PRJNA596481. Read data for transcriptomics are available under BioProject PRJNA600972. RNAseq data, WGCNA modules, and enrichment analyses can be found in supplemental files File S1 (ant data) and File S2 (fungus data). Additional results and discussions are presented in File S3. Supplemental material available at figshare: https://doi.org/10.25387/g3.12121659.

## Results & Discussion

### Manipulated behavior of Camponotus floridanus after laboratory infections

We set out to identify parasite and host genes involved in manipulated biting and clinging behavior observed in *Ophiocordyceps*-infected *Camponotus* ants. To this end, we infected *C. floridanus* with *O. camponoti-floridani* to compare gene expression levels in this host-parasite interaction with those published for *C. castaneus* and *O. kimflemingiae* (de Bekker *et al.* 2015).

**Figure 2:**
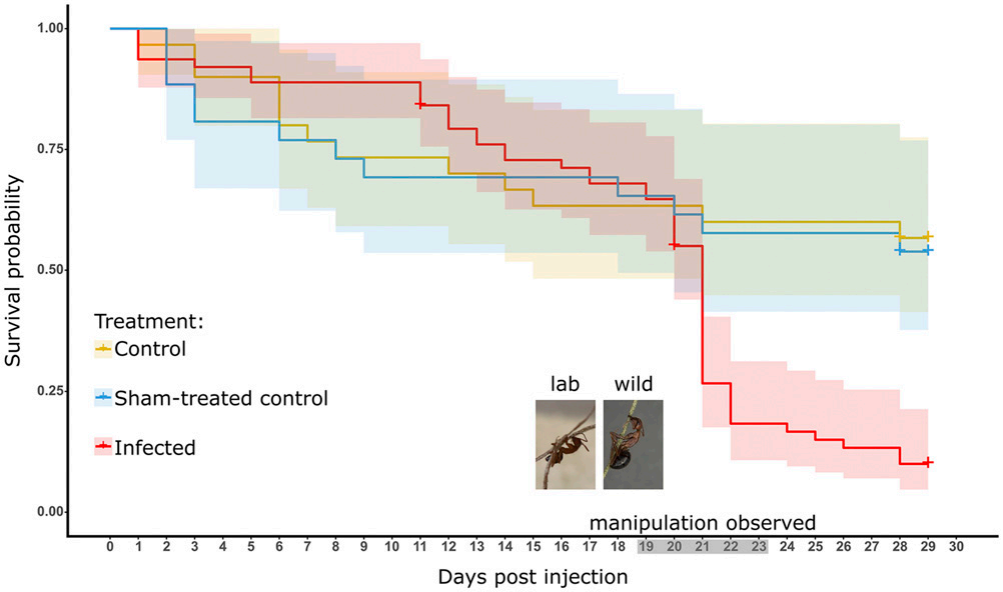
Survival curves of infection experiment ants. We introduced three treatment groups in the infection experiments, ants not injected (yellow, control n = 30), injected with media-only (blue, sham-treated control n = 26), or injected with the *O. camponoti-floridani* (red, infected n = 63). Shaded regions around survival data indicate 95% confidence intervals. Treatment had a significant effect on survival (*P* = 0.00059, log-rank test). Manipulations were observed only in infected ants, 19 to 23 dpi (gray axis shading). Crosses indicate censorship events when ants were removed from the experiment and no longer contributed to analysis. The photo inserts show a live lab infected ant (left) and a wild manipulated cadaver (right).

All manipulated *C. floridanus* ants (n = 11) clung to plants with their legs, with two individuals additionally biting the plant. This lab-infected manipulation phenotype well-approximated wild manipulations (Figure 2). Live manipulated ants commonly displayed subtle tremors, feeble clasping motions, and low responsiveness to puffs of air. If these ants fell from their manipulation perches, they continued clasping motions but otherwise did not right themselves or move (n = 2). Overall survival was significantly different based on treatment (*P* = 0.00059, log-rank test), with the 95% confidence interval of infected ants lower and not overlapping with sham treated or control ants by 21 dpi (Figure 2).

Manipulated clinging and biting behavior displayed by *O. camponoti-floridani* infected *C. floridanus* ants occurred within a stereotypic dpi-window with apparent time of day synchronization. Infected ants displayed manipulated behavior starting 19 dpi, with the last manipulation at 23 dpi (Figure 2). All observed manipulations occurred pre-dawn from at least as early as ZT 20 until ZT 23 (*i.e.*, 4 hr to 1 hr before lights-on). When we captured the onset of manipulated clinging and allowed the ant to progress to death, the time between manipulation and death was 0.5 hr to 2 hr (n = 3, of six dead manipulated ants). In previous infection studies with *O. kimflemingiae*, manipulations of *C. castaneus* occurred 16 through 24 dpi and shortly after subjective dawn (ZT 3), which was the first daily observation period of that study (de Bekker *et al.* 2015). Infected *C. castaneus* usually died at least 5 hr after manipulation. Such stereotypic patterns have also been reported for other ant-manipulating *Ophiocordyceps*, both in the laboratory (Sakolrak *et al.* 2018) and in the wild (Hughes *et al.* 2011).

Our opportunistic preliminary field observations have found live manipulated *C. floridanus* one to three hours after solar noon (n = 4). This is out of phase from our laboratory observations for this species, as well as those made for *O. kimflemingiae*-infected *C. castaneus* (de Bekker *et al.* 2015). Differences in abiotic factors, such as light, temperature, and humidity, across labs and field observations could have led to these phase shifts (Hughes *et al.* 2011; Andriolli *et al.* 2019; Cardoso Neto *et al.* 2019). Therefore, rather than selecting a ZT at which to sample infected *C. floridanus* for RNAseq, we sampled based on behavioral phenotypes comparable to infected *C. castaneus*: immediately upon observing manipulated clinging behavior or death after manipulation. We expect sampling according to phenotype instead of daily timing to have produced more comparable gene expression profiles across the two species-interaction studies.

A role for light-cues in the summiting aspect of *Ophiocordyceps* manipulation of ants has previously been proposed (Chung *et al.* 2017; Andriolli *et al.* 2019). Insect manipulating baculovirus strains also induce summiting behavior in silkworm hosts, with an apparent phototactic element in coordinating manipulation (Kamita *et al.* 2005; van Houte *et al.* 2014a; Han *et al.* 2018). Baculovirus may only require light before, but not during summiting (Han *et al.* 2018). Such light-coordinated behavior, rather than direct phototaxis, possibly underlies the pre-dawn summiting we observed in the laboratory.

### De novo hybrid assembly of the Ophiocordyceps camponoti-floridani genome

The reliable alignment and separation of mixed sequencing reads to determine relative abundances of both host and parasite transcripts requires high-quality reference genomes of both organisms (Figure 1). A recently updated genome of *C. floridanus* is publicly available (Shields *et al.* 2018). To generate a high-quality *O. camponoti-floridani* genome, we combined Nanopore long-reads and Illumina short-reads data in a hybrid assembly.

After polishing and contaminant removal steps, our *de novo* hybrid assembly contained 13 contigs encompassing 30.5 Mbp, with a N50 of 3.8 Mbp and 53x Nanopore coverage (Table 1). Pezizomycotina telomeric repeats, TTAGGG (Podlevsky *et al.* 2008), are present on nine of the contigs, three of which are bounded on both ends by repeats and therefore should represent whole chromosomes of 5.6 Mbp, 3.8 Mbp, and 2.5 Mbp in length. One of the 13 contigs represents a putative mitochondrial genome of 272,497 bp. We identified this mitochondrial contig by *(i)* its high read coverage (735x) compared to the genome average (53x), *(ii)* its low GC content (27%) compared to the total assembly (48%), *(iii)* the presence of homologs to known mitochondrial proteins on this contig but nowhere else in the genome (BLASTp of ATP6, COB, COX1, and NAD1 of *Aspergillus niger*) (Joardar *et al.* 2012), and *(iv)* its circular sequence structure. The genome assembly appeared nearly complete with 99.7% of pezizomycotina benchmarking universal single-copy orthologs (BUSCOs) (v 3, using OrthoDB v. 9) (Simão *et al.* 2015) (Table 1) (GenBank Accession JAACLJ000000000).

**Table 1:** camponoti-floridani genome assembly

Assembly characteristic	Value	Annotation	Number of genes
contigs	13	BLAST	6291
size (Mbp)	30.5	PFAM	5460
N50 (Mbp)	3.8	GO	3515
largest contig (Mbp)	5.6	SignalP	801
predicted genes	7455	SSP	271
BUSCO%	99.7	TMHMM	1372
GC%	48.4	2° metabolism	111
Telomeric repeat areas	12	MEROPS	243
		transcription factor	206

Genome annotations identified 7455 gene models (Table 1). Most genes received functional annotations based on PFAM domains (73%) or BLAST descriptions (86%). We also identified 801 putatively secreted proteins containing a SignalP domain (11%) and 271 small secreted proteins (SSPs, 3.6%). Only 19% of the SSPs carried known PFAM domains and only 37% returned BLAST descriptions. With many SSPs lacking clear functional annotations, these SSPs may contain a pool of novel bioactive compounds secreted by *O. camponoti-floridani*.

### RNAseq identifies differentially expressed genes associated with manipulation

To discover candidate fungal and ant genes that underpin the manipulated behavior of *O. camponoti-floridani*-infected *C. floridanus* hosts, we sequenced the transcriptomes of samples obtained before, during, and after manipulation. Ant heads collected during and after manipulation contained mixed transcriptomes of both host and parasite (Figure 1). The average number of aligned reads for each fungal and ant transcriptome fell between 11.6M and 41.5M, except for the ant transcriptome of the dead manipulated samples (*i.e.*, after manipulation), which resulted in only 4.9M aligned reads (Figure 1). For both fungal culture and healthy ant head control samples, 93% of the RNAseq reads aligned to their respective reference genomes (Shields *et al.* 2018). We aligned 57% and 74% of reads obtained from live manipulated or dead manipulated ant heads to the *O. camponoti-floridani* genome, respectively. In contrast, these samples only resulted in 35% and 17% of reads that aligned to the *C. floridanus* genome (Figure 1) (Shields *et al.* 2018). These findings corroborate previous findings in other *Ophiocordyceps*-ant interactions that the fungus has colonized the ant head by the time of manipulation and rapidly destroys host tissue for its own growth as the host dies (Hughes *et al.* 2011; de Bekker *et al.* 2015).

To validate differential expression analysis between our biological sample groups, we first determined if the variation between replicates within these groups was smaller than the variation between them. Unsupervised dendrograms based on replicate ant and fungal gene expression profiles indeed clustered biological replicates together (Figure S2). However, the fungal profile of live manipulation sample 4 (L4) was placed ambiguously relative to live and dead manipulated samples (Figure S2A). Regardless, we did not exclude sample L4 as an outlier, as we are not confident about the confines of typical disease progression and gene expression during this time point. As these samples were selected on observed behavioral phenotype, the transcriptional state of genes essential for manipulation is plausibly shared despite differences in other genes.

We included all samples to identify differentially expressed genes correlated to control (fungal culture and healthy ants), live manipulation, and dead manipulation samples, finding 1431 ant and 2977 fungal genes differentially expressed between at least one pair of sample conditions. To identify genes that are plausibly involved in *Ophiocordyceps* manipulation of ant behavior, we performed various complementary gene expression analyses for both fungal and ant genes, which are detailed below.

### Principal component analyses identify host changes linked to manipulation

**Figure 3:**
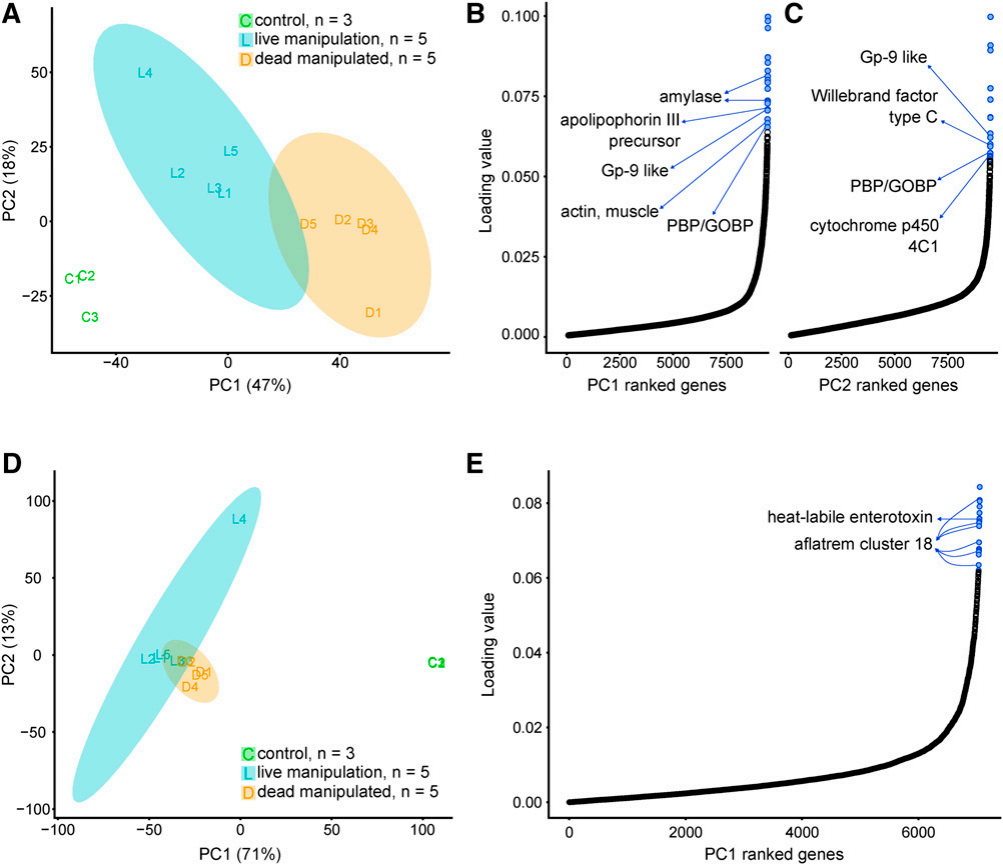
Principal component analyses and principal component loading value plots of RNAseq data. Analyses are based on normalized ant (A, B, C) and fungal (D, E) gene expression values. PCA plots (A, D) show the relationship between samples that serve as controls (green) and those that were collected during live manipulation (blue) and after host death (orange). Shaded regions indicate 95% confidence ellipses. (A.) Replicates of host gene expression vary across PC1 as the state of the host progresses from healthy control ants to live manipulated hosts to dead manipulated hosts killed by the fungus. PC2 primarily describes the variation between healthy controls and live manipulated ants, but all biological groups vary along this axis. (B, C.) All ant genes ranked by loading values in PC1 (B) or PC2 (C). Most genes have low loading values, however a relatively high-loading value subset contribute the most to PC1 or PC2. Of these high value genes, the top 20 include genes that may play key roles during infection and manipulation (blue). (D.) Gene expression of the fungal parasite interacting with the ant host is clearly distinguished from that of fungal culture control samples by PC1. (E.) All fungal genes ranked by loading values in PC1. The top 20 fungal PC1 genes are highlighted and include toxin related genes (blue).

Through PCAs we identified the genes that contributed the most to the transcriptome variations between our biological groups. An unsupervised PCA of all normalized ant transcriptome data distinguished host gene transcription prior to infection (control), during manipulation (live manipulation), and after (dead manipulated) (Figure 3A). Principal Component 1 explained 47% of the variation between host transcriptional profiles over progression of the infection from healthy to manipulated to dead ants. To identify the major contributors to PC1, we ranked and plotted ant genes by their PC1 loading values (Figure 3B, Table S1). The top 20 genes of PC1 included genes putatively related to odor detection (pheromone-binding protein Gp-9-like and a pheromone-binding protein (PBP)/general odorant-binding protein (GOBP) family PFAM domain-containing gene), nutrition and energy balance (alpha-amylase 1, alpha-amylase A-like, and an apolipophorin-III precursor PFAM domain-containing gene), and muscle tissue (myogenesis-regulating glycosidase-like and muscle actin). These genes were also found to be differentially expressed between sample types and are further highlighted in the DEG section below.

PC2 largely described the variation between healthy control ants and live manipulated hosts, explaining 18% of sample variation in total. The top 20 PC2 genes shared PBP/GOBP domain genes with PC1. However, the PC2 top 20 also included putative DEGs involved in insect immunity (defensin and a von Willebrand factor type C domain-containing gene) and insect starvation response-mediated by juvenile hormone (JH) (cytochrome P450 4C1-like) (Figure 3C, Table S2).

### Principal component analyses identify fungal effectors produced during infection

A PCA of all normalized fungal transcriptome data generated a PC1 explaining 71% of the transcriptional variation. PC1 indicated a large separation between transcription profiles prior to infection (control) and after (live or dead manipulated) (Figure 3D). Fungal gene expression profiles from live and dead manipulated ants were less different from each other, as indicated by the partial overlap of their 95% confidence ellipses (Figure 3D). The second principal component (PC2, 13%) primarily described the difference between replicate L4, and other fungal samples, which the sample dendrogram also indicated (Figure S2). Major elements of PC1 likely indicated genes linked to infection, manipulation, and killing of the host.

The top 20 of all fungal genes, as determined by their PC1 loading values (Figure 3D, Table S3), peaked during live manipulation in both *O. camponoti-floridani* and *O. kimflemingiae* (de Bekker *et al.* 2015). Although significantly higher expressed compared to culture, their expression relative to dead host samples was not always significantly different. This set of 20 genes contained multiple candidates of interest identified in secondary metabolite clusters and as DEGs. These genes, discussed in more detail in the sections below, included multiple members of a putative aflatrem biosynthesis pathway (cluster 18) and a putative enterotoxin that was extremely highly upregulated in both *O. camponoti-floridani* and *O. kimflemingiae*.

### Weighted gene coexpression network analysis correlates parasite gene expression during manipulation to host gene networks

We also analyzed ant and fungal RNAseq data using a WGCNA to describe coexpressed gene networks (modules) correlated with control, live manipulated, and dead manipulated samples. This analysis shows which genes may be expressed in concert with each other and how those networks correlate to the manipulation state of the host or coexpressed networks in the parasite. We then characterized these networks based on annotation enrichment analyses.

For ant gene coexpression patterns, the WGCNA generated 22 modules, which we named A1 – A22. Expression of four modules, A4, A5, A6, and A10, were significantly positively correlated with live manipulation (*P* ≤ 0.05, Fisher’s asymptotic test on Pearson correlation values) and either had a negative or no significant correlation with control and dead samples (Figure S3). Four additional modules, A14, A15, A17, and A18, were significantly negatively correlated to live manipulation. Of these modules, A17 and A18 were also positively correlated to healthy control ants. Taken together, the WGCNA identified eight ant gene modules with significant correlations to the time of live manipulation that highlight transcriptional responses to fungal infection and manipulation (File S1).

To investigate general functions of the ant gene modules, we performed enrichment analyses of annotated PFAM domains and GO terms present in those modules. In module A4, genes putatively involved in proteasome activity and odorant detection (*i.e.*, PBP/GOBP PFAM domain) were overrepresented (File S1). These gene functions were also highlighted by the ant PCA above (Figure 3B-C). Reduced detection of odor cues and related social interactions have been hypothesized to play a role in the early stages of manipulation that precede biting by *Ophiocordyceps*-infected carpenter ants (de Bekker *et al.* 2015). Modules A5, A6, and A10 were enriched for annotations related to gene and DNA regulatory processes (File S1).

**Figure 4:**
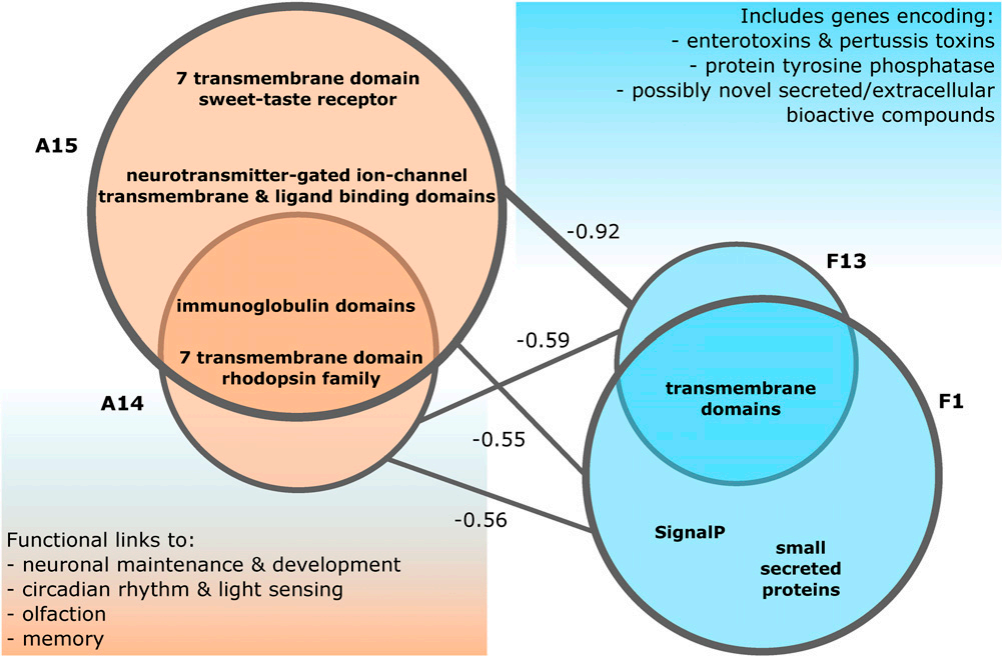
Ant WGCNA modules correlated with fungal modules suggest interference with neuronal function. A14 and A15 are enriched for PFAM domains that suggest neuronal functions, which are negatively correlated to fungal modules F1 and F13 that are enriched for extracellular and secretion signals. These fungal modules also contain putative effectors discussed in greater detail in following sections, such as enterotoxins or protein tyrosine phosphatase (PTP). Pearson correlation values shown by lines connecting modules.

In the ant modules negatively correlated to live manipulation, we detected gene modules associated with neuronal function. A14 and A15 had a 7 transmembrane receptor (rhodopsin family) PFAM enrichment related to light sensing and cellular signaling (Table S4). This suggests a loss in light sensitivity as a mechanism to promote light-seeking behavior, which has previously been hypothesized to occur in manipulated ants prior to biting to assure light levels that promote fungal growth and transmission (Andriolli *et al.* 2019). Module A15 additionally contained an overrepresentation of genes with a 7 transmembrane domain (sweet-taste receptor) related to glutamate or GABA receptors and neurotransmitter gated ion channels (*i.e.*, putative acetylcholine, glycine, and glutamate receptors) (File S1). Both modules were also predominantly enriched for transmembrane transport, ion regulation, and cell signaling activity annotations, with multiple immunoglobulin domain enrichments (File S1). Although, the underlying genes for these enrichments were generally not DEGs. The immunoglobulin domain overrepresentations contained a variety of genes putatively encoding cell-surface binding proteins related to neuronal development, maintenance, and activity, such as IgLON family proteins with additional light sensing or circadian, olfaction, and memory related functions (Table S5). These multiple signals tied to the ant’s neurobiology in modules A14 and A15 suggest disrupting neuronal function underscores manipulated behavior (Figure 4). Genes from enriched annotations point to mechanisms dysregulating light responsiveness and neurotransmitters related to behavior and muscle activity (Figure 4, Table S4, Table S5).

**Figure 5:**
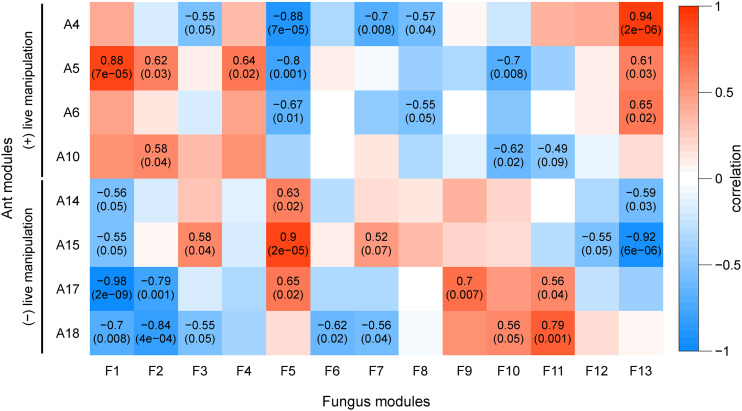
WGCNA of fungal and ant modules correlated to each other. Fungal gene expression (columns) show correlation (color and top number in each cell) and p-value (in parentheses) of gene modules to selected ant modules that are either positively or negatively correlated to samples during live manipulation (rows). Pearson correlation and p-values (Fisher’s asymptotic test) are shown only for significant module to module correlations. Modules F1, F2, and F13 are enriched for secretion signals or transmembrane domains and therefore may contain extracellular fungal effectors. Modules A14 and A15 have PFAM domain overrepresentations suggesting a role in neuronal function and development.

The WGCNA for fungal gene coexpression patterns generated 13 modules, F1 – F13, and correlated them to the three possible sample types – control culture, live manipulation, and death after manipulation (File S2). Three modules, F1, F2, and F4, were significantly positively correlated with live manipulation. F1 and F2 were additionally negatively correlated with fungal growth in control culture (Figure S4). Subsequently, we performed an additional WGCNA with all 13 fungal modules against the eight ant modules that had significant correlations to live manipulation of the host to describe possible behavioral changes and responses to infection. We used these eight ant modules as a new set of trait data (*i.e.*, eigengenes) to correlate our fungal modules to (Figure 5). Using this strategy rather than separately associating fungal and ant gene networks to the broader categories of our biological groups, we aimed to make a more detailed connection between fungal gene expression and the corresponding transcriptional changes in the host.

**Figure 6:**
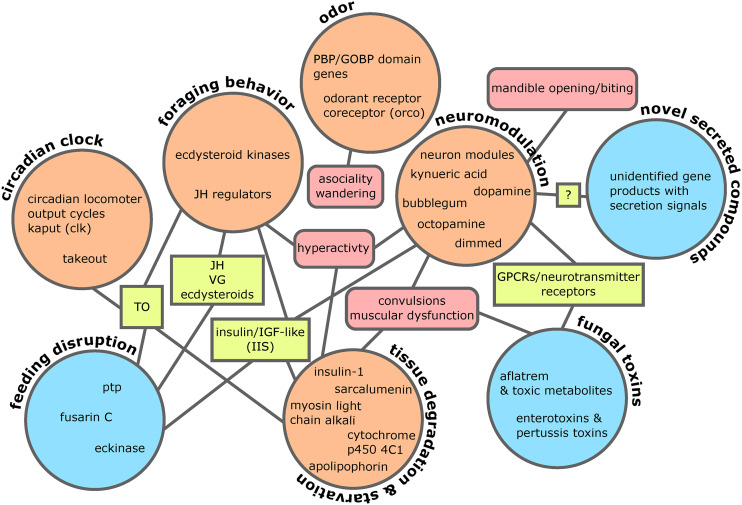
Major themes and candidate manipulation mechanisms. These genes and pathways are possible players in manipulation, emerging from RNAseq of *O. camponoti-floridani-C. floridanus* and comparisons to published *O. kimflemingiae-C. castaneus* interactions (de Bekker *et al.* 2015). Ant DEGS and proposed mechanisms (orange circles) are connected to fungal counterparts (blue circles) via shared molecular players (yellow rectangles) and phenotypes (red rounded-rectangles).

Fungal modules F1, F2, and F13 appeared to be major contributors to fungal effects on the ant host as these modules were positively correlated to activation of manipulation-associated ant modules and negatively correlated to ant modules deactivated during manipulation (Figure 4, Figure 5). Putatively secreted genes (*i.e.*, SignalP and SSP annotations) were overrepresented in F1 and F2, as were transmembrane domains (TMHMM) in modules F1 and F13. These enrichments suggest that these fungal modules are involved in extracellular interactions with the host. Oxidation-reduction related annotations and transcription factors were also overrepresented in module F2. Oxidation-reduction terms are a hallmark of parasite-host interactions and were overwhelmingly found in *O. kimflemingiae* – *C. castaneus* interactions (de Bekker *et al.* 2015). Although fungal modules F1 and F13 did not harbor any other annotation enrichments, they indicated an increase of putatively secreted effectors and virulence related activity in *O. camponoti-floridani* in correspondence to the changing expression of ant gene networks correlated with manipulation. Notably, F1 and F13 negatively correlated with ant modules A14 and A15, which in turn appear to be associated with neuron function (Figure 4). Therefore, modules F1 and F13 possibly contain extracellular fungal effectors that dysregulate neuron function and health (Figure 6).

### Differentially expressed ant genes during infection and manipulation

To consider genome-wide enrichment patterns in ant gene expression in relation to infection, manipulation, and host death, we divided our RNAseq data into subsets representing different interpretations of gene function that may underlie host responses and effects of fungal activity (Figure 1). Genes that were significantly upregulated from control ants to live manipulation and then downregulated once the ant died may indicate specific responses to infection and active behavioral manipulation by the parasite (120 genes, File S1). We also analyzed genes that were upregulated during live manipulation from control and then had sustained or increased transcription until the ant died (88 genes, File S1). Being upregulated, these genes plausibly play a role during manipulation but may also be more generally associated with infection or host death. Similarly, we considered differentially expressed gene sets that were downregulated at live manipulation relative to both healthy ants and dead hosts (6 genes, File S1), and those holding or dropping further in dead hosts (529 genes, File S1). We collected our samples for the dead manipulated time point under frequent observation, such that these ants should reflect a manipulated ant transcriptome just as the host dies. As many significant transcriptional effects would likely lag behind the moment of presumed death, we expect to have captured the state of a moribund ant as it dies. To propose host changes that could be underlying the manipulated behaviors in *C. floridanus*, we closely investigated the functional annotations of these DEG sets and compared them to those previously found for manipulated *C. castaneus*.

#### Host gene expression patterns related to tissue destruction and nutrition:

The destructive invasion and consumption of host tissues by *Ophiocordyceps* may have implications for behavior beyond mere host death. Notably, the ants’ muscles are affected, leading to muscle atrophy and hypercontraction that have been suggested to have a role in the locked biting position of manipulated ants (Hughes *et al.* 2011; Fredericksen *et al.* 2017; Mangold *et al.* 2019). In line with this aspect of the disease, a putative sarcalumenin gene was downregulated during live manipulation in both *C. floridanus* and *C. castaneus* (threefold and eightfold decrease from control to live manipulation, respectively) (de Bekker *et al.* 2015). Sarcalumenin has been shown to interact with calcium and regulate muscle excitation and fatigue in mammalian systems (Zhao *et al.* 2005; O’Connell *et al.* 2008). Additionally, an essential component of myosin motor proteins, a myosin light chain alkali (Yamashita *et al.* 2000), was downregulated in both species of ant hosts during live manipulation (fourfold and twofold decrease from control to live manipulation in *C. floridanus* and *C. castaneus*, respectively). A gene BLAST annotated as “actin, muscle”, was a DEG in both ant species, and we found additional muscle genes downregulated in *C. floridanus* (File S3). Taken together, gene expression levels in heads of both host species during manipulated biting and clinging show hallmarks of muscle tissue destruction and dysregulation (Figure 6).

Over the course of infection by *Ophiocordyceps*, ant hosts are expected to lose energy stores to the parasite. The expression of genes related to nutritional balance will likely reflect this change in physiology. Moreover, starvation often induces hyperactivity in animals exhibited by increased levels of locomotion behavior (Yang *et al.* 2015), which in some cases could be adaptive for the host (Hite *et al.* 2019) or reflect parasitic manipulation. Indeed, we found DEGs putatively related to primary metabolism and nutrition in *Ophiocordyceps*-infected ants, which we frequently observed to display elevated bouts of locomotion prior to death (*i.e.*, ELA). Two putative apolipophorin genes (apolipophorin and an apolipophorin III precursor domain containing gene) were significantly downregulated over the course of infection and manipulation in both *C. floridani* and *C. castaneus* (between twofold to ninefold decrease from control to live manipulation) (de Bekker *et al.* 2015). Apolipohorin III has immune functions that operate in a trade-off manner with metabolism of high fat diets in insects (Adamo *et al.* 2010).

Beyond signs of a diminishing lipid metabolism (File S3), we detected differential cytochrome p450 4C1 expression, which has been implicated in starvation responses in cockroaches and responds to JH treatments in females (Lu *et al.* 1999). In cockroaches, starvation upregulated this gene, while we detected thirty putative cytochrome p450 4C1 genes downregulated from control to live manipulation in *C. floridanus*, with three significantly downregulated homologs in *C. castaneus* (de Bekker *et al.* 2015) (File S3). An alpha amylase A-like gene, involved in the degradation of complex sugars (Da Lage 2018), was additionally downregulated over the course of infection (286-fold decreases from control to death in *C. floridanus* and 12-fold in *C. castaneus*). This indicates a reduction of starch metabolism in the likely starving host. Also tied to nutritional state, we observed a putative *insulin-1* gene downregulated in both species of ant hosts (threefold and sixfold decrease from control to live manipulation in *C. floridanus* and *C. castaneus*, respectively).

Insulin/insulin-like growth factor (IGF) signaling (IIS) pathways have been implicated in behavior, division of labor, and establishment of reproductive caste and associated behaviors in ants and bees (Ament *et al.* 2008; Chandra *et al.* 2018). Juvenile hormone and vitellogenin (VG) appear to play an important role in IIS pathways and behavior, although typically the strongest effects are observed during development and early life (Bloch *et al.* 2000; Brent *et al.* 2006; Lengyel *et al.* 2006; Nelson *et al.* 2007; Ament *et al.* 2008, 2010; Velarde *et al.* 2009; Penick *et al.* 2011; Dolezal *et al.* 2012; Libbrecht *et al.* 2013; Corona *et al.* 2013, 2016; Das 2016; Chandra *et al.* 2018; LeBoeuf *et al.* 2018; Opachaloemphan *et al.* 2018). Nutritional status, energy demands, and behaviors such as foraging appear to be interlinked in these eusocial insects. In our transcriptome dataset, we found two JH activating genes (juvenile hormone acid O-methyltransferases) and two deactivating genes (juvenile hormone epoxide hydrolases) that were significantly downregulated in live manipulated ants compared to healthy controls (Shinoda and Itoyama 2003; Zhang *et al.* 2005). A similar gene expression pattern was found for a homologous JH epoxide hydrolase in *C. castaneus* (de Bekker *et al.* 2015). *Ophiocordyceps*-induced changes in ant behavior could be partially due to, or reflected in, the dysregulation of ant JH levels (Figure 6) and IIS functions (File S3).

Ecdysteroids can indirectly influence insect development and behavior by interacting with JH (Libbrecht *et al.* 2013). Modification of ecdysteroids has been implicated in the behavioral manipulation of moth larvae by baculovirus to assure that they remain in an elevated position (Hoover *et al.* 2011; Ros *et al.* 2015; Han *et al.* 2015). We detected an enrichment of genes putatively encoding ecdysteroid kinases in the subset of ant genes that were downregulated from control to live manipulation and remained lowly expressed or decreased further into host death. The modification of ecdysteroids could, thus, also be involved in *Ophiocordyceps* manipulation of ant behavior to induce climbing behavior, and perhaps be a more general mechanism underlying summiting in parasite-manipulated insects. Possible fungal effectors interacting with these JH or ecdysteroid pathways are discussed in more detail in the differentially expressed fungal gene section below (Figure 6).

#### Shifts in circadian rhythms and clock-controlled genes that regulate behavior:

The cooption and manipulation of circadian rhythms have been proposed as an underlying mechanism for synchronized biting and the disruption of exploratory foraging behaviors in manipulated *Camponotus* (de Bekker *et al.* 2015, 2017b; de Bekker 2019). Healthy ants display daily regimented foraging behaviors controlled by the molecular clock. These behaviors are seemingly disrupted and replaced by manipulated climbing, biting, and clinging behaviors that in turn take place in a synchronized manner (Hughes *et al.* 2011; de Bekker *et al.* 2015). As controls and manipulated ants were time-matched within this study and within de Bekker *et al.* (2015), differential expression of clock-related genes is likely due to infection by *Ophiocordyceps* and not an artifact of time of day during sampling. In line with the circadian clock hypothesis, we found that the core clock gene, circadian locomotor output cycles kaput (*clk*) (Darlington *et al.* 1998), was significantly downregulated from healthy control to live manipulated ants in both *C. camponoti-floridani* (10-fold decrease) and *C. castaneus* (threefold decrease) (de Bekker *et al.* 2015). Additionally, a gene putatively encoding the clock-controlled Takeout (TO) protein showed a similar gene expression pattern, again both in *C. floridanus* and *C. castaneus* (threefold and fourfold decreases from control to live manipulation, respectively) (de Bekker *et al.* 2015). Takeout is a JH interacting protein involved in insect foraging behaviors and starvation response (Sarov-Blat *et al.* 2000; Meunier *et al.* 2007; Schwinghammer *et al.* 2011) and has been proposed as a possible target for parasitic disruption of insect locomotor activity and behavior (van Houte *et al.* 2013). We have found more evidence for this hypothesis and further discuss possible fungal effectors dysregulating *to* in the fungal DEG section below (Figure 6).

#### Dysregulation of odor detection:

Odor detection is at the basis of social organization and behavior in ants, mediated by multiple odorant receptors and odorant binding proteins. Pheromone binding proteins are a subset of odorant binding proteins specialized in binding pheromones and as such play a vital role in an individual’s response to external stimuli (van den Berg and Ziegelberger 1991; Chang *et al.* 2015). We found 16 odor receptor and binding protein genes differentially expressed in *C. floridanus* (File S1, File S3), 13 of which were differentially expressed between controls and live manipulation, six being downregulated and seven upregulated.

One of the odorant receptors, putatively encoding an odorant receptor coreceptor (Orco), is highly conserved in insects and has a central role in odor detection (Jones *et al.* 2005; Stengl and Funk 2013; Zhou *et al.* 2014; Lin *et al.* 2015). Dysregulation of *orco* in ants has been linked to changes in overall sensitivity to odorants, and affects behavior such as time spent outside the nest, ability to detect prey, and aggression toward conspecifics (Yan *et al.* 2017; Ferguson *et al.* 2020). One of two putative *orco* genes in *C. floridanus* was significantly upregulated in the ant during live manipulation compared to control (threefold increase). Its homolog in *C. castaneus* was significantly downregulated (fourfold decrease) (de Bekker *et al.* 2015). However, up- or downregulation of *orco* may lead to similar phenotypes as both agonist and antagonist effects on Orco are reported to produce similar changes in *C. floridanus* nestmate recognition (Ferguson *et al.* 2020). Similarly, in both ant host species, multiple genes putatively encoding PBP Gp9 were differentially expressed during manipulation (de Bekker *et al.* 2015) (File S3) and have been implicated in mediating fire ant colony social dynamics (Ross 1997; Ross and Keller 1998; Krieger and Ross 2002; Gotzek and Ross 2007; Gotzek *et al.* 2007). *Ophiocordyceps* infected individuals may be unable to properly communicate with nestmates and recognize organizational signals due to disrupted odorant reception. This dysregulation could be facilitating the wandering behaviors we observed in infected individuals and prove to be parasite-adaptive if infected ants are thereby more commonly positioned in suitable fungal transmission sites (Figure 6).

#### Dysregulation of neurotransmitter signaling:

Dysregulation of neurotransmitter and neuron-modulating compounds are a plausible parasite strategy to manipulate host behavior. We identified a suite of ant neuron regulating and neurotransmitter receptor genes in the WGCNA modules that were negatively correlated to samples collected at live manipulation (see above and Figure 4). Closely inspecting DEGs, we also identified putative ant neuromodulatory compounds that were differentially expressed over the course of infection.

Dysregulation of kynurenic acid, an anticonvulsant and neuroprotective neuroinhibitor, has been implicated in mammalian neurodegenerative disease, changes in activity levels, and reduced motor coordination (Yu *et al.* 2004b, 2006). A putative kynurenine/alpha-aminoadipate aminotransferase, which promotes the synthesis of kynurenic acid, was upregulated in the ant during live manipulation (threefold increase from control to live manipulation in *C. floridanus* and 33-fold increase in *C. castaneus*) (de Bekker *et al.* 2015). Additionally, metabolomics on manipulated *C. castaneus* identified that *O. kimflemingiae* secreted the neuroprotectant ergothionine (Loreto and Hughes 2019). Indeed, neural tissues appear to be among the last host tissues to be severely degraded (Hughes *et al.* 2011; Fredericksen *et al.* 2017). The preservation of neural tissue by compounds such as parasite ergothionine or host kynurenic acid is potentially critical for manipulation by fungal effectors operating via changes in ant biogenic amines and the disruption of neuron functions (Figure 6).

Biogenic monoamines have neuromodulatory roles in insects, and changes in monoamine activity and synthesis may underlie manipulated phenotypes in ants. Acting through G-protein coupled receptors (GPCRs), octopamine functions as a neurotransmitter in insects modulating learning and memory, foraging behavior, starvation-induced locomotion activity, insulin levels, olfactory decision making, aggression, and social interactions (David and Verron 1982; Schulz *et al.* 2002; reviewed in Roeder 2005; Yang *et al.* 2015; Li *et al.* 2016). Such processes may serve as targets for *Ophiocordyceps* to induce behavioral modifications such as ELA (Hughes *et al.* 2011; de Bekker *et al.* 2015). Moreover, parasitoid venom-induced hypokinesia in cockroaches has been linked to modulation of octopamine activity levels, most likely through manipulation of octopamine receptors (Libersat and Gal 2014). Consistent with this scenario, we identified octopamine receptors that were differentially expressed between live manipulated and control ants. In *C. floridanus*, a putative octopamine receptor beta-2R was downregulated during manipulation (twofold decrease from control to live manipulation), while beta-3R was found to be upregulated in *C. castaneus* (sixfold increase from control to live manipulation) (de Bekker *et al.* 2015). This suggests that octopamine responsiveness is dysregulated in *Ophiocordyceps*-infected ants, although the specific mechanisms used by different fungal species may differ.

Dopamine, another biogenic monoamine, also functions as a neurotransmitter in insects and regulates motor neuron activity, locomotion behavior, and biting behavior, among other processes, sometimes in a clock-controlled fashion (Cooper and Neckameyer 1999; Ceriani *et al.* 2002; Szczuka *et al.* 2013; reviewed in Yamamoto and Seto 2014). Tyrosine 3-monooxygenase drives the rate-limiting step in dopamine synthesis (Daubner *et al.* 2011). We found homologous genes putatively encoding for this enzyme to be significantly upregulated during manipulation in *C. floridanus* (fourfold increase from control to live manipulation) and *C. castaneus* (threefold increase) (de Bekker *et al.* 2015). In addition, a putative DOPA decarboxylase, which catalyzes the final step of dopamine synthesis (Daubner *et al.* 2011) was significantly upregulated in *C. floridanus* during manipulation (threefold from control to live manipulation). Changes in dopamine levels may also be implicated in immune function, as a precursor to melanin, which is a component of ant immunity (Ratzka *et al.* 2011). However, dysregulation related to both octopamine and dopamine in ants during manipulation indicated a role for biogenic monoamines in producing the observed behaviors (Figure 6).

In addition to neuroprotective agents and biogenic monoamines, we identified two more differentially expressed genes that could be involved in aberrant neuronal functioning in manipulated individuals. Both *C. floridanus* and *C. castaneus* exhibited reduced expression of a putative *dimmed*-like transcription factor in live manipulated ants compared to the healthy controls (sixfold and ninefold decrease, respectively) (de Bekker *et al.* 2015). The downregulation of *dimmed* resulted in the dysregulation of neuropeptide secretion and IIS-responsive neuronal maintenance in *Drosophila* (Hamanaka *et al.* 2010; Luo *et al.* 2013; Liu *et al.* 2016). A putative *bubblegum* gene was also downregulated in *C. floridanus* during manipulation (fivefold decrease from control to live manipulation) as was the homolog in *C. castaneus* (fourfold decrease) (de Bekker *et al.* 2015). In *Drosophila*, *bubblegum* mutants displayed neurodegeneration, retinal degeneration, and reduced locomotor activity (Min and Benzer 1999; Sivachenko *et al.* 2016).

### Differentially expressed putative fungal effector genes

As for the ant gene expression data, we divided the fungal data into subsets representing different interpretations of gene function in relation to manipulation (Figure 1). Fungal genes that were significantly upregulated from culture to live manipulation and then downregulated once the host died likely played a role in infection and/or behavioral manipulation (307 genes, File S2). For enrichment analysis, we further narrowed this set of upregulated genes to the top 50^th^ percentile of genes with the largest downregulation in dead hosts (168 genes, File S2). Genes in this 50^th^ percentile were tightly regulated relative to the manipulation event and, therefore, possibly the most manipulation specific genes in our dataset. We also considered genes that were upregulated from culture to live manipulation and then had sustained or increased transcription in the dead host (1088 genes, File S2). Being upregulated, these genes plausibly play a role in infection or manipulation. They may also play a role in fungal activities associated with host death, such as killing the host and consuming dead host tissues. Similarly, we considered differentially expressed gene sets that were downregulated at live manipulation relative to both culture and dead hosts (61 genes, File S2), a 50^th^ percentile strongly down subset (33 genes, File S2), and downregulated from culture and holding or dropping further in dead hosts (867 genes, File S2). These gene sets could either indicate genes not important for manipulation, or the reduced transcription of inhibitors. The two fungal species shared more homologs in upregulated (29%) than downregulated (18%) DEGs. Upregulated DEGs also contained more hits for pathogenicity in the Pathogen-Host Interaction database (Urban *et al.* 2017) (S3 File). This suggested that upregulation during manipulation contains proportionally more genes with conserved function and fitness constraints, *i.e.*, involvement in infection and manipulation.

Over 25% (239 genes) of genes with SignalP secretion signals were upregulated from culture to living manipulated ants. Similarly, 22% (195 genes) were upregulated in the putative secretome of *O. kimflemingiae* (de Bekker *et al.* 2015). The increased activation of the *Ophiocordyceps* secretome during manipulation by both species suggests a critical role for secreted compounds in modifying host behavior. This is in line with microscopy evidence demonstrating that fungal cells do not grow invasively into the ant’s brain (Hughes *et al.* 2011; Fredericksen *et al.* 2017), but rather likely manipulate behavior peripherally by secreting neuroactive compounds. Yet, only 54% of the *O. camponoti-floridani* secretome upregulated from culture to manipulation had PFAM annotations (Table S6), leaving about half of these potential key players without an assigned putative function.

#### Ophiocordyceps upregulated GPCR-interfering toxins during manipulation:

Many cellular receptors, including neurotransmitter receptors, are GPCRs that could serve as targets for fungal ADP-ribosylating toxins (Figure 6). Heat-labile enterotoxins are in this class and have been described for pathogens such as *Escherichia coli*, *Cordyceps bassiana*, and *Metarhizium robertsii* (reviewed in Lin *et al.* 2010; Mannino *et al.* 2019). Heat-labile enterotoxins of *E. coli* transfer an enzymatic domain into host cells to modify GTP-binding proteins and interfere with GPCRs and subsequent intracellular signaling through increased cyclic AMP levels. This process eventually leads to cell dysfunction and apoptosis (reviewed in Lin *et al.* 2010; Mangmool and Kurose 2011). Other microbial toxins that disrupt GPCRs or intracellular signaling showcase pathogenic effects that suggest toxins could benefit *Ophiocordyceps* in infecting and manipulating host ants. Heat-stable bacterial enterotoxins dysregulate pheromone production in insect fat bodies (Wiygul and Sikorowski 1986, 1991). The ADP-ribosylating mosquitocidal toxin of *Bacillus sphaericus* acts on G proteins and has lethal effects on mosquitoes (Thanabalu *et al.* 1991). Cytotoxic necrotizing factor-1 interacts with GTPases and contributes to *E. coli* invasion of central nervous tissues and crossing of the blood-brain-barrier in mammals (Khan *et al.* 2002).

**Figure 7:**
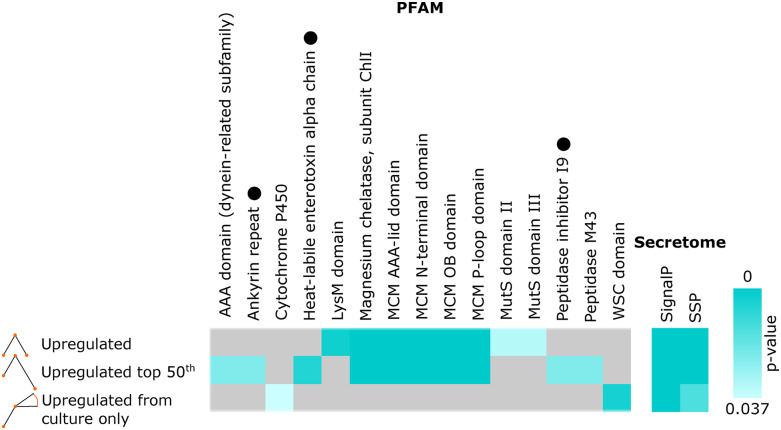
PFAM and secretion signal domain enrichments in DEG sets with increased transcription during live manipulation. “Upregulated” refers to genes with peak transcription during live manipulation. “Upregulated top 50^th^ ” refers to the subset of upregulated genes with the strongest subsequent downregulation in dead manipulated samples. “Upregulated from culture only” are genes with increased transcription from culture to live manipulation, but exhibited no change or increasing transcription with host death. PFAMs associated with genes that have possible roles in manipulation include: Heat-labile enterotoxin alpha chain, Ankyrin repeat, and Peptidase inhibitor I9 (indicated by black dots), and Peptidase M43 (File S3).

The *O. camponoti-floridani* genome contains 35 predicted heat-labile enterotoxin genes based on the PFAM annotation Enterotoxin_a, which was enriched among upregulated genes during manipulation (Figure 7). This group of upregulated enterotoxins also resulted in enrichment of the GO terms “multi-organism process,” “interspecies interactions between organisms,” “toxin activity,” and “pathogenesis” (File S2). Thirty putative enterotoxins carried SignalP secretion domains, 10 of which were upregulated from culture to live manipulation, and then six were sharply downregulated in the dead host. The most strongly upregulated enterotoxin in *O. camponoti-floridani* displayed a >12,000-fold increase in transcripts from culture. The putative ortholog in *O. kimflemingiae* displayed a marked > 3,000-fold upregulation (de Bekker *et al.* 2015). Moreover, this enterotoxin gene appears to be exclusively conserved in ant-manipulating *Ophiocordyceps* species (de Bekker *et al.* 2017a), suggesting a potential specialized role in facilitating ant manipulation. This enterotoxin gene was also present in the manipulation associated fungal WGCNA module F2 with seven other enterotoxins. Module F1 contained an additional three enterotoxin genes.

Ant-infecting *Ophiocordyceps* genomes are enriched for heat-labile enterotoxins compared to generalist fungal pathogens and have been suggested to play a major role in *Ophiocordyceps* pathogenesis. For example, *O. kimflemingiae* has 36 putative enterotoxins, *Ophiocordyceps australis* has 20, and *Ophiocordyceps polyrhachis-furcata* has 22 (Wichadakul *et al.* 2015; de Bekker *et al.* 2017a), while the generalist entomopathogens *C. bassiana*, *M. robertsii*, and *Isaria javanica* have 13, six, and five enterotoxins, respectively (Xiao *et al.* 2012; Mannino *et al.* 2019; Lin *et al.* 2019). The number of putative enterotoxin genes, their notable upregulation, and membership in manipulation associated gene modules strongly suggest a role for these toxins during *Ophiocordyceps* infection and manipulation (Figure 6).

Other putative ADP-ribosylating toxins were also upregulated in *O. camponoti-floridani* at the time of manipulation relative to growth in culture and in dead hosts. This included two *Bordetella pertussis* toxin A genes that were also identified in the manipulation correlated fungal WGCNA module F1. These genes carried Ankyrin repeat domains, which contributed to the enrichment for this PFAM annotation among genes upregulated during manipulation (Figure 7). One of these pertussis toxins was upregulated in both fungal species over the course of infection (*i.e.*, fourfold increase from culture to live manipulation in *O. camponoti-floridani*, and 538-fold in *O. kimflemingiae*). The active sites of pertussis toxins are similar to heat-labile enterotoxins and also act via GPCR interference (Locht *et al.* 1986), and therefore may have comparable effects on host physiology.

#### Fungal serine proteases are upregulated during manipulated biting behavior:

Fungal subtilases are subtilisin-like serine proteases that have been implicated in entomopathogenic interactions by degrading insect cuticle (chitin). During infection, *Metarhizium anisopliae* produces increased levels of serine protease Pr1, leading to host death. Additionally, Pr1 overproducing strains of *M. anisopliae* decrease host-feeding and injections of Pr1 are toxic (St Leger *et al.* 1996). Often, these proteases contain a peptidase inhibitor I9 domain, which is present in propeptides and assists folding and activation once cleaved (reviewed in Figueiredo *et al.* 2018).

Inhibitor I9 domain encoding genes were found to be enriched among genes upregulated during manipulation (Figure 7). Two of these three I9 containing genes were subtilases with MEROPS S8A annotations similar to Pr1 found in *C. bassiana* and *M. anisopliae* (Joshi *et al.* 1995). We found six S8A annotated *O. camponoti-floridani* genes upregulated during manipulation relative to culture (some lack I9 domains, File S3), one of which was found in WGCNA module F1. Similarly, six serine proteases were upregulated during manipulation in *O. kimflemingiae* (de Bekker *et al.* 2015). If Ophiocordyceps fungi employ these subtilases for virulence in general or use them for manipulation specifically is not clear. However, the highly species-specific ant manipulating Ophiocordyceps carry fewer subtilases (O. camponoti-floridani n = 16, O. kimflemingiae n = 18, and other *Ophiocordyceps* species n < 20) compared to generalist entomopathogenic fungi (*C. bassiana* n = 43, *Cordyceps militaris* n = 26, *M. anisopliae* n = 55, and *Metarhizium acridum* n = 43) (Gao *et al.* 2011; Zheng *et al.* 2011; Xiao *et al.* 2012; de Bekker *et al.* 2015; Wichadakul *et al.* 2015). A larger repertoire of proteolytic enzymes may facilitate infection of various host species and is thought to be associated with a broader host range in fungal entomopathogens (Xiao *et al.* 2012; Lin *et al.* 2019). The relatively low number of subtilases in *Ophiocordyceps* genomes corroborates their high species-specificity (de Bekker *et al.* 2014b; Araújo *et al.* 2018; Sakolrak *et al.* 2018).

#### A putative fungal eckinase may modulate host ecdysteroids:

We have uncovered evidence for changes in ant JH, IIS pathways, and ecdysteroids being linked to modified behavior. One possible scenario is that fungal effectors are targeting these elements of host physiology directly. Ecdysteroids have been implicated in viral manipulation of caterpillars that display a summit disease phenotype. Baculovirus secretes an enzyme, EGT, which inactivates an ecdysone molting hormone and alters larval feeding behavior and development (O’Reilly 1995). In certain species of caterpillar, *egt* is implicated in driving fatal summit disease, while it only alters pre-molting climbing behavior in others (Hoover *et al.* 2011; Ros *et al.* 2015; Han *et al.* 2015). Moreover, the entomopathogenic fungus *Metarhizium rileyi* appears to attack host ecdysteroid pathways by secreting an enzyme that disrupts larval development (Kiuchi *et al.* 2003; Kamimura *et al.* 2012).

In *O. camponoti-floridani*, we detected a phosphotransferase gene with eckinase and SignalP domains that was significantly higher expressed during manipulation than in culture (twofold increase). This gene was also present in WGCNA module F2. Eckinase activity helps mediate the balance of active free ecdysteroids and inactive storage forms (Sonobe *et al.* 2006). As such, *O. camponoti-floridani* could be utilizing a similar strategy as baculovirus or *Metarhizium* to modify its host, targeting ecdysteroid levels with an ecdysteroid modulating enzyme (Figure 6).

#### Upregulated protein tyrosine phosphatase implicated in insect hyperactivity and ELA:

Also implicated in baculovirus infection, PTP has a suggested role in ELA phenotypes of infected caterpillars (Kamita *et al.* 2005; Katsuma *et al.* 2012), but not summiting (van Houte *et al.* 2014b). Whether enzymatic activity of PTP is needed for modifying caterpillar behavior appears to differ by the study system used (Katsuma *et al.* 2012; van Houte *et al.* 2012). However, when enzymatic activity is critical, PTP has been hypothesized to act via changes of TO or the cGMP-dependent serine/threonine protein kinase, Foraging (For) (van Houte *et al.* 2013). The protein For underlies feeding behaviors in ants and other insects (Osborne *et al.* 1997; Ben-Shahar *et al.* 2002, 2003; Lucas and Sokolowski 2009; Ingram *et al.* 2011) and serves as an intriguing candidate for fungal disruption. Circadian expression and phototactic effects of *for* have previously been shown (Ben-Shahar *et al.* 2003; Ingram *et al.* 2011). Therefore, if fungal PTP could dysregulate activity of For in ants, this would be a plausible strategy for the parasite to alter when and how long the ant host leaves the nest to engage in foraging behaviors and locomotor activity. Similarly, disruption of TO, which is downregulated in manipulated *Camponotus*, could dysregulate foraging behavior (Figure 6).

What function PTP may have for fungal manipulation of *Camponotus* ants is uncertain at this time. However, *O. camponoti-floridani* has seven putative *ptp* genes, of which five were upregulated from culture during manipulation, with three of those putatively secreted. We found four *ptp* genes in manipulation WGCNA modules F1 (n = 1) and F2 (n = 3), and two of these upregulated *ptp* genes in *O. camponoti-floridani* (365- and eightfold increase from culture to manipulation) have homologs in *O. kimflemingiae* that were upregulated in similar fashion (28- and twofold increase) (de Bekker *et al.* 2015).

### Fungal secondary metabolites involved in manipulation and infection

We identified 25 secondary metabolite clusters in the *O. camponoti-floridani* genome and determined the bioactive compounds they produce from gene annotations. The genomes of *O. kimflemingiae* and *O. polyrhachis-furcata* contain comparable numbers of annotated clusters, 25 and 24, respectively (de Bekker *et al.* 2015; Wichadakul *et al.* 2015). Notably, 23 of the *O. camponoti-floridani* clusters contain genes with homologs identified previously in *O. kimflemingiae* as secondary metabolite cluster genes (de Bekker *et al.* 2017a). Their gene expression patterns and functional annotations offer insights into the possible fungal secondary metabolites involved in manipulation and infection as discussed below and File S3.

#### Cluster 18, Manipulation-related aflatrem-Like indole-diterpene alkaloid production:

**Figure 8:**
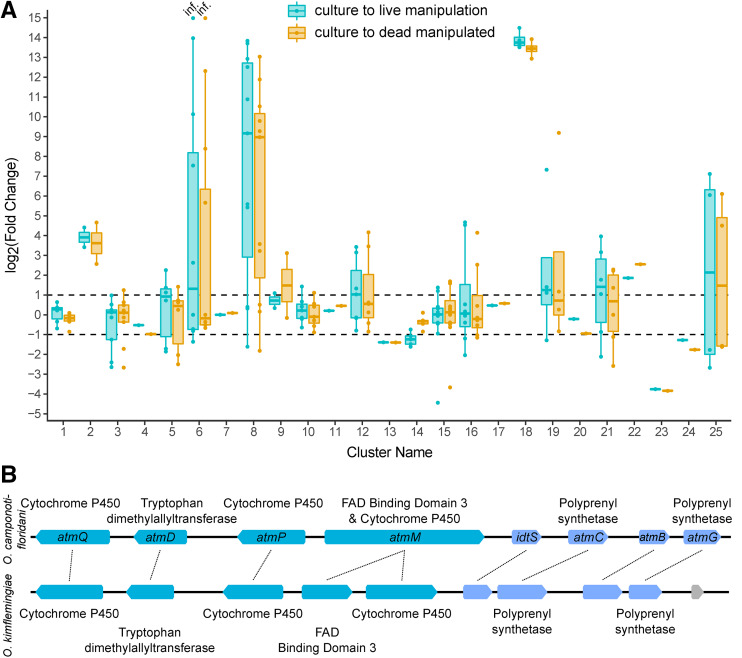
Changes in expression of secondary metabolite cluster genes relative to control conditions. (A.) Fold change of secondary metabolite cluster genes transcripts during live-manipulation (blue) and in dead-manipulated samples (orange) relative to control culture. Dots represent fold change of a single gene within a metabolite cluster. Dashed lines indicate a twofold change in transcript abundance (*i.e.*, log_2_(Fold Change) = ±1). Clusters 2, 6, 8, 12, and 18 are possibly involved in pathways producing entomopathogenic compounds similar to aflatoxin, fusarin C, aflatoxin, citrinin (File S3), and aflatrem, respectively. Many genes of these clusters also displayed notable increases in transcript levels relative to culture. Points labeled “inf.” in cluster 6 have infinite fold increases due to culture RPKM values equal to 0. (B.) Schematic of cluster 18 genes (top) and corresponding homologs in *O. kimflemingiae* (bottom). PFAM annotations are labeled outside the depicted loci and, for *O. camponoti-floridani*, BLASTp hits for aflatrem synthesis proteins are indicated inside. Teal indicates cluster genes identified bioinformatically, blue genes are selected cluster-adjacent genes based on their similar expression, putative function, and proximity to the main cluster (not represented in [A.]). The locus in gray is unrelated to the cluster. Image modified from output generated by Geneious Prime (v 2019.0.3, Biomatters).

Metabolite cluster 18 putatively produces an alkaloid that appears to be a mycotoxin. The entirety of this eight-gene cluster demonstrated a striking upregulation during infection (Figure 8A), as did the homologous cluster in *O. kimflemingiae* (de Bekker *et al.* 2015). Additionally, six cluster 18 genes were found in fungal manipulation WGCNA module F2. These clusters are highly similar in gene composition and organization between the two *Ophiocordyceps* species (Figure 8B) (de Bekker *et al.* 2015). Previously, de Bekker *et al.* (2015) identified one possible product of this cluster to be an ergot alkaloid based on a tryptophan dimethylallyltransferase (TRP-DMAT) backbone gene of the cluster (> 12,000 fold increase from culture to live manipulation in *O. camponoti-floridani*, 5,900 fold in *O. kimflemingiae*).

However, homology searches indicated that cluster 18 is more likely to produce an aflatrem-like indole-diterpene alkaloid (Figure 8B). Aflatrem is a neurotoxic tremorgen that causes “stagger disease” in poisoned hosts and is closely related to other mycotoxins such as paxilline or lolitrem (Gallagher and Hawkes 1986). By interfering with big potassium (BK) channels, and, gamma amino butyric acid (GABA)-ergic and glutamatergic processes, aflatrem can induce muscle tremors, changes in activity level, and confusion (Valdes *et al.* 1985; Gant *et al.* 1987; Yao *et al.* 1989; Knaus *et al.* 1994). *Ophiocordyceps* infected ants display comparable symptoms such as convulsions (Hughes *et al.* 2011), muscle hypercontraction (Mangold *et al.* 2019), and atypical exploratory behaviors (de Bekker *et al.* 2014b) (Figure 6).

Adjacent to secondary metabolite genes in cluster 18, we identified additional putative cluster members based on similar expression patterns, functional annotations related to secondary metabolism, and proximity (approximately within 3 kbp of another cluster gene, *i.e.*, double the distance between any genes in the original cluster). Among these genes, we identified an upregulated terpene cyclase *atmB*-like gene. This gene has a homolog in *O. kimflemingiae* annotated as *paxB* (the corresponding paxilline synthesis gene compared to aflatrem), which suggests that aflatrem or a similar indole-diterpene is synthesized by cluster 18 (Figure 8B).

We subsequently performed a BLASTp of translated *Aspergillus flavus* aflatrem synthesis genes (atmA, atmB, atmC, atmD, atmG, atmM, atmP, and atmQ) (Nicholson *et al.* 2009) against the entire *O. camponoti floridani* genome. All proteins except AtmA scored BLASTp hits with the lowest E-value hits in or adjacent to cluster 18. All putative aflatrem synthesis homologs were present in a single cluster of the *O. camponoti-floridani* genome, while these genes are split between two genomic sites in *A. flavus* (Nicholson *et al.* 2009).

Despite not detecting any homologous proteins to AtmA in the *O. camponoti-floridani* genome, there is an upregulated gene in the cluster-adjacent set that has a BLAST annotation corresponding to *idtS*, but otherwise lacks a GO or PFAM annotation (Figure 8B). Described in the lolitrem-producing fungi *Epichloë* and *Neotyphodium* as necessary for indole-diterpene synthesis (Schardl *et al.* 2013), the molecular role of IdtS is not entirely clear. Similarly, the role of AtmA in aflatrem synthesis has not yet been fully elucidated (Nicholson *et al.* 2009). The *O. camponoti-floridani idtS* homolog may work in concert with the indole-diterpene synthesis genes identified in cluster 18, although we currently do not fully understand its function.

Aflatrem gene cluster synteny and upregulation during manipulation were well conserved between *O. camponoti-floridani* and *O. kimflemingiae* (Figure 8B), suggesting an important role for this mycotoxin in facilitating manipulation. Metabolite clusters are suggested to reflect ecological and evolutionary pressures and allow insight into possible critical functions for the life history of a fungus (Slot and Gluck-Thaler 2019). Possible fitness benefits are suggested by these genes being clustered at a single genomic site and their high degree of conservation between *O. camponoti-floridani* and *O. kimflemingiae*. Such organization may allow these fungi to better regulate aflatrem genes in tandem and maintain them as a physically linked genetic unit, unlike *A. flavus* for example, which carries these genes at two distinct sites.

#### Clusters 8 and 2, Putative aflatoxin production during infection and manipulation:

Aflatoxin is a potent mycotoxin and carcinogen with lethal effects on insects (Trienens and Rohlfs 2011). We predicted the polyketide metabolite clusters 8 and 2 to produce an aflatoxin or similar metabolite, based on the polyketide synthase (PKS) backbone gene in these clusters having a starter unit:acyl carrier protein transacylase (SAT) domain that facilitates the first step in the synthesis of aflatoxins (Brown *et al.* 1996). Upregulation of most genes in putative aflatoxin-like clusters 8 and 2 during live manipulation suggests a role for this mycotoxin during the final stages of infection (eight of 11 and two of two genes upregulated, respectively) (Figure 8A). Five genes from cluster 8 were in the manipulation associated WGCNA module F2. The PKS backbone of cluster 8 was upregulated during manipulation in both *O. camponoti-floridani* and *O. kimflemingiae* (*i.e.*, 43-fold increase from culture to live manipulation in *O. camponoti-floridani*, 4,350-fold in *O. kimflemingiae*). The PKS backbone of cluster 2 was also upregulated (11-fold increase from culture to live manipulation). However, the cluster 2 backbone homolog in *O. kimflemingiae* were downregulated during this time.

Using the *Aspergillus parasiticus* aflatoxin gene cluster (Yu *et al.* 2004a), we performed a BLASTp search of 25 proteins against the *O. camponoti-floridani* genome. All but two genes (*aflI* and *aflX*) had at least one *O. camponoti-floridani* homolog. The PKS backbone of cluster 8 is homologous to *aflC*, the PKS of the *A. parasiticus* cluster (E-value = 2.61e-98). Additionally, cluster 8 contained the top BLASTp hit for *aflJ*. Furthermore, *aflD* returned a low E-value hit within the cluster (1.94e-06), but fell short of our bit score 50 cutoff (46.21). Although these results do not demonstrate that cluster 8 produces aflatoxin, they do suggest a role in generating aflatoxin or a similar compound.

The PKS backbone of cluster 2 is also a putative homolog to AflC (E-value = 1.38e-93) and the cluster’s O-methyltransferase was the top BLASTp hit for AflP, which generates the penultimate product in aflatoxin synthesis (Yu *et al.* 2004a). Although our bioinformatic approach only identified these two genes in cluster 2, similarly regulated adjacent genes could generate products consistent with the aflatoxin synthesis pathway. In proximity to cluster 2, the *O. camponoti-floridani* genome contains an upregulated gene encoding a protein homologous to AflQ, known to play an important role in the final step of aflatoxin synthesis (Yu *et al.* 2004a). We also identified a possible homolog to *aflT* (E-value = 4.22e-38), which is known to be involved in aflatoxin synthesis (Yu *et al.* 2004a). A homolog of *A. parasiticus* Velvet-complex member *laeA* is also adjacent to the cluster, which functions as a regulator of the aflatoxin-intermediate sterigmatocystin (Bok and Keller 2004). The top BLASTp hit for AflR, which is a transcription factor involved in aflatoxin synthesis and interacts with LaeA in a regulatory feedback loop (Yu *et al.* 2004a; Bok and Keller 2004) was found on the same contig, albeit distantly from cluster 2. Also possibly interacting with LaeA, two velvet domain containing genes were upregulated from culture to manipulation, one of which has a similarly upregulated homolog in *O. kimflemingiae*. The homologs of *alfC*, *alfP*, *alfT*, and a velvet transcription factor were also found in fungal WGCNA module F2.

Taken together, metabolite clusters 2 and 8 and their homologs in *O. kimflemingiae* suggest that both species of *Ophiocordyceps* have the capacity to produce an aflatoxin-like compound. However, contrasting transcriptomics data for cluster 2 across these species indicated that the regulation of aflatoxin production during infection and manipulation might be different across these species.

#### Cluster 6, Fusarin C as a possible virulence or behavior modifying factor:

Fusarin C is the likely product of cluster 6, consisting of nine genes, two of which were upregulated during live manipulation (Figure 8A). Three other genes in this cluster were also present in WGNCA modules F1 (n = 1) and F2 (n = 2). Fusarin C is a carcinogenic mycotoxin and although produced by the entomopathogen *Metarhizium anisopliae*, insecticidal or antibacterial activity appears to be absent without culturing additives (Krasnoff *et al.* 2006). Additionally, insects possibly resist some effects of fusarin C by detoxification pathways that respond to xenobiotic and toxic challenges (Gelderblom *et al.* 1988; Gui *et al.* 2009; Rohlfs and Churchill 2011). However, fusarin C may have non-lethal effects as suggested by its apparent mycoestrogen activity, demonstrated in mammalian cells (Sondergaard *et al.* 2011). Estrogens could be involved in the production of ecdysteroids and VG with effects on development, reproduction, and diapause in insects (reviewed in Das 2016). Speculatively, exogenous estrogen activity from fusarin C produced by *Ophiocordyceps* could, thus, be disrupting ant physiology and behavior (Figure 6).

Four genes appear necessary for fusarin C synthesis in *Fusarium fujikuroi*: *fus1*, *fus2*, *fus8*, and *fus9* (Niehaus *et al.* 2013). All four homologs in cluster 6 represented the top or sole BLASTp result in the *O. camponoti-floridani* genome. Two additional fusarin C cluster proteins, Fus3 and Fus4 (Niehaus *et al.* 2013), also had top BLASTp hits in cluster 6. Only *fus4* (sevenfold increase from culture to live manipulation) and *fus9* (0 RPKM to 2 RPKM) homologs were upregulated during manipulation, while the other cluster genes were either not differentially expressed or downregulated. *Ophiocordyceps kimflemingiae* and *O. polyrhachis-furcata* also contain clustered homologs of the necessary fusarin C synthesis genes (de Bekker *et al.* 2015; Wichadakul *et al.* 2015). In the *O. kimflemingiae*, all but the *fus1* homolog were upregulated during live manipulation relative to both culture and dead hosts (de Bekker *et al.* 2015).

Similar to our findings for aflatoxin, the presence of homologous fusarin C clusters among *Ophiocordyceps* species suggests that all utilize fusarin C-like mycoestrogens, but when this metabolite is produced or used may differ between species. Possibly, fusarin C has a role earlier in infection when subtler behavioral changes such as altered locomotor activity manifest. Changes in ecdysteroid or VG related behaviors, such as time spent foraging or nest occupation, may be plausible effects of the introduction of such a mycoestrogen and the underlying synthesis genes may no longer be strongly expressed during the final manipulation stage in all *Ophiocordyceps*.

### Proposed effector-response interactions and conclusions

A growing body of literature has proposed possible fungal effectors of the ant-manipulating *Ophiocordyceps* based on genomic, transcriptomic, and metabolomic analyses (de Bekker *et al.* 2014b, 2015, 2017a; Wichadakul *et al.* 2015; Kobmoo *et al.* 2018). Although phylogenetic and experimental evidence indicate that *Ophiocordyceps*-ant interactions are species specific (Kobmoo *et al.* 2012; de Bekker *et al.* 2014b; Araújo *et al.* 2018; Sakolrak *et al.* 2018), we hypothesized that *Ophiocordyceps* species share mechanisms to infect and manipulate their hosts as they face similar host and transmission related challenges (Chetouhi *et al.* 2015; Loreto *et al.* 2018). Indeed, we have found transcriptomic and genomic signals during *Ophiocordyceps camponoti-floridani* manipulation in line with previous work on *O. kimflemingiae* and other species, indicating several possible common contributors to infection and manipulation. Not only did these putative mechanisms of infection and manipulation emerge from comparisons across species of both parasite and host, they were consistent among the analyses that we employed (PCA, WGCNA, and DEG analysis).

Gene expression changes related to neuron function in manipulated ants could be an effect of secreted fungal compounds (Figure 6). Neurotransmitter receptors present one of many possible GPCR targets for fungal ADP-ribosylating toxins. Additionally, a putative aflatrem alkaloid toxin may be attacking the host nervous system, inducing stagger disease-like symptoms. A wealth of unannotated putative secreted proteins also offers candidate manipulation genes that may act on the nervous system, or other aspects of host biology.

Behavior regulating pathways involving players such as TO, CLK, JH, ecdysteroids, and IIS may be intertwined with each other and respond to fungal disruption via mechanisms such as PTP, eckinases, or fusarin C (Figure 6). In particular, foraging behaviors may be modified and result in ELA and wandering behaviors of manipulated ants that facilitate dispersal of infected hosts to transmission sites. In line with dysregulation of task performance, changes in ant odorant reception and chemical communication with nestmates could contribute to similar asocial wandering phenotypes.

Taken together, we propose several fungal candidate manipulation genes and possible ant behavioral pathway responses that could drive manipulated climbing, biting, and clinging behaviors in *Ophiocordyceps*-manipulated individuals. However, the candidates that we identified must still be functionally tested for a more precise understanding of how, and if, they play a critical role in the manipulation of host behavior. Similarly, follow-up metabolomic approaches would validate the production of secondary metabolites by the putative gene clusters we propose here. Investigating potential protein-level mimicry by the parasite to interfere with host processes and physiology may be fruitful as well (Hebert *et al.* 2015). The comparison of multiple *Ophiocordyceps* and their respective ant hosts could identify possible species-specific mimicry that would indicate tight coevolutionary relationships and precise mechanisms of manipulation. Such studies could eventually be expanded to other parasitic manipulation systems since we find signatures of potentially convergently evolved mechanisms across manipulators (*i.e.*, baculovirus). As such, our study provides a springboard toward deeper functional and evolutionary understandings of the molecular mechanisms underlying host manipulation. In turn this could offer additional insights into novel bioactive compounds and the neurobiology of animal behavior in general.
